# Searching for protein partners of short-chain 3-hydroxyacyl-CoA dehydrogenase (SCHAD) reveals keratin 8 as a novel candidate for interaction in pancreatic β-cells

**DOI:** 10.1186/s12860-025-00544-w

**Published:** 2025-06-05

**Authors:** Kelly Velasco, Janniche Torsvik, Johanna L. St-Louis, Sarah Baghestani, Jonas S. G. Silvander, Rohit N. Kulkarni, Diana M. Toivola, Anders Molven

**Affiliations:** 1 Gade Laboratory for Pathology, Department of Clinical Medicine, University of Bergen, Bergen, Norway; 2 Department of Pathology, Haukeland University Hospital, Bergen, Norway; 3 Islet Cell and Regenerative Biology, Joslin Diabetes Center, Harvard Medical School, Boston, MA USA; 4 Cell Biology, Faculty of Science and Engineering, Åbo Akademi University, Turku, Finland; 5 InFLAMES Research Center, Åbo Akademi University, Turku, Finland; 6 Department of Medicine, Beth Israel Deaconess Medical Center, Harvard Medical School, Boston, USA; 7 Harvard Stem Cell Institute, Boston, USA; 8 Section for Cancer Genomics, Haukeland University Hospital, Bergen, Norway

**Keywords:** Congenital hyperinsulinism of infancy, Short-chain 3-hydroxyacyl-CoA dehydrogenase, SCHAD, *HADH*, Intermediate filaments, Keratin 8, *KRT8*, Yeast 2-hybrid screening, Proximity ligation assay

## Abstract

**Background:**

Short-chain 3-hydroxyacyl-CoA dehydrogenase (SCHAD) is a ubiquitously expressed mitochondrial enzyme with a role in the degradation of fatty acids. Because the protein also is a negative regulator of insulin secretion in pancreatic β-cells, inactivating mutations in the SCHAD gene (*HADH*) cause congenital hyperinsulinism of infancy (CHI) and severe hypoglycemia. Here we sought to identify novel interaction partners of SCHAD that might be particularly relevant for the endocrine pancreas.

**Results:**

Employing the SCHAD protein as bait, we performed yeast 2-hybrid screening of a cDNA library made from human islets of Langerhans. Surprisingly, the screening revealed the intermediate filament protein keratin 8 (K8) as a putative interaction partner of SCHAD with very high confidence. Previous reports have linked K8 to glucose homeostasis, and we confirmed the SCHAD interaction by co-immunoprecipitation in HEK293 cells. SCHAD and K8 expression were then characterized in the human β-cell model EndoC-βH1. By using proximity ligation assay, we demonstrated that stimulating the cells with a high level of glucose triggered a transient increase in the interaction. However, when studying knockout mice, we found that the loss of either K8 or SCHAD did not change the expression level of the other interaction partner. Still, when K8 knockout mice were challenged with a ketogenic diet, upregulation of SCHAD expression was blunted compared to the upregulation observed in wildtype littermates.

**Conclusions:**

We propose that the SCHAD protein interacts with K8 in a way that might be relevant for proper functioning of the pancreatic β-cell. Whether the SCHAD-K8 interaction influences the phenotype of CHI remains to be demonstrated.

**Supplementary Information:**

The online version contains supplementary material available at 10.1186/s12860-025-00544-w.

## Introduction

Congenital hyperinsulinism (CHI) refers to genetic disorders characterized by persistent hypoglycemia secondary to inappropriately elevated insulin secretion. These disorders can have a monogenic or syndromic origin, and according to the underlying molecular mechanism, they may be classified in three categories: channelopathies, metabolopathies and transcription factor defects [1, 2].

The metabolopathies comprise enzyme anomalies and other metabolic defects [3]. One such form of CHI is caused by deficiency of the ubiquitously expressed, mitochondrial enzyme short-chain 3-hydroxyacyl-CoA dehydrogenase (SCHAD), encoded by the *HADH* gene [4–6]. SCHAD has two different roles in metabolism: one as catalyst of the third of four steps in the fatty acid β-oxidation pathway, and a second as negative regulator of insulin secretion from the pancreatic β-cells [7]. Consistent with the latter function, both global and β-cell-specific knockout of SCHAD in mice lead to hypoglycemia that is exacerbated by amino acids [8, 9]. Pull-down assays using human and mouse tissues, as well as enzyme kinetics studies in mouse islet homogenates, have shown that SCHAD interacts with and inhibits glutamate dehydrogenase (GDH), another ubiquitously expressed mitochondrial enzyme [8, 10]. Notably, activating mutations in the gene coding for GDH can also trigger CHI [11]. Thus, the current model of how *HADH* mutations cause CHI, posits that they lead to a deficiency of SCHAD protein and therefore a failure to inhibit GDH in the β-cells. The overly active GDH results in an increased level of ATP within the β-cell, which again is followed by inappropriately stimulated secretion of insulin [8].

Different approaches to studying SCHAD protein-protein interactions have revealed multiple metabolic interaction partners, some of which appear to be tissue-specific and extra-mitochondrial [8, 12]. This led to the hypothesis that SCHAD exerts non-enzymatic functions in metabolic super-complexes that integrate multiple metabolic pathways [12]. However, to the best of our knowledge, a search for interacting partners of SCHAD has not been conducted in pancreatic islets, an investigation that could be of relevance to a comprehensive understanding of how insulin secretion is regulated. In the present study, our aim was therefore to identify SCHAD protein interactions that might be involved in the regulation of insulin secretion. To this end, we performed yeast two-hybrid (Y2H) screening in a human islet cDNA library using the SCHAD protein as bait.

The hit with highest confidence score was the *KRT8* gene, which encodes the epithelial intermediate filament protein officially denoted ‘keratin, type II cytoskeletal 8’ or simply keratin 8 (K8) [13]. In β-cells, this protein forms a filamentous network in complex with keratin 18 (K18), with structural and functional effects when K8 is missing [14–16]. We evaluate the finding from the Y2H screen by various approaches and present evidence for an as yet undescribed interaction between SCHAD and K8.

## Materials and methods

### Yeast two-hybrid screening

The screening was performed by Hybrigenics Services, Evry-Courcouronnes, France (www.hybrigenics-services.com). Coding sequence from the human *HADH* gene (NCBI reference NM_005327.4), covering either amino acids 13–314 (Bait 1) or amino acids 13–214 (Bait 2) of the SCHAD protein, was PCR-amplified and cloned into the pB27 vector as a C-terminal fusion to LexA (LexA-HADH). The two constructs were sequenced for quality control and used as baits to screen a random-primed cDNA library from human islets of Langerhans, constructed into the pP6 vector. The pB27 and pP6 vectors derive from the original pBTM116 [17] and pGADGH [18] plasmids, respectively. Two hundred million clones (20-fold the complexity of the library) for Bait 1 and 93 million clones (9-fold the complexity) for Bait 2 were screened using a mating approach with YHGX13 (Y187 ade2-101::loxP-kanMX-loxP, matα) and L40ΔGal4 (mata) yeast strains as previously described [19]. By selection on a medium lacking tryptophan, leucine and histidine, 441 His + colonies for Bait 1 and 352 His + colonies for Bait 2 were identified. The prey fragments of the positive clones were amplified by PCR and sequenced at their 5’ and 3’ junctions. The resulting sequences were used to identify the corresponding interacting proteins in the GenBank database (NCBI) using a fully automated procedure. A confidence score (predicted biological score, PBSc) was attributed to each interaction as described previously [20].

### Plasmids and cell culture

Generation of SCHAD knockout (SCHADKO) HEK293 cells and plasmids for eukaryotic expression of V5/His-tagged SCHAD protein have been previously described [6]. Regular HEK293 cells (ATCC CRL-1573) and SCHADKO HEK293 cells were cultured in DMEM medium (Gibco, catalogue number 41966-029) supplemented with 10% FBS (Gibco, 10270-106) and PenStrep (Sigma-Aldrich, P4458), and maintained in 5% CO_2_ at 37 °C. EndoC-βH1 cells (Human Cell Design) were cultured in OPTIβ complete culture medium (Human Cell Design) in 100 mm dishes coated with βCoat (Human Cell Design) as described [21]. All cell lines were tested for Mycoplasma infection every two weeks using the MycoAlert Mycoplasma detection kit (Lonza; LT07-318).

### Antibodies

Monoclonal antibodies were used to detect K8 by western blotting and immunofluorescence. Mouse anti-K8 M20 (Thermo Fisher Scientific, MA1-06318) was employed against the human protein and rat anti-K8 Troma I (DSHB) against the mouse protein. V5-tagged SCHAD in transfected cells was detected using mouse anti-V5 (Thermo Fisher Scientific, R960-25). On western blots, SCHAD was detected using either of two polyclonal rabbit antibodies (Atlas antibodies, HPA039588; GeneTex, GTX105167).

Secondary HRP-conjugated anti-IgG antibodies for western blots were from Thermo Fisher Scientific (goat anti-mouse, 62-6520; goat anti-rabbit, 65-6120; goat anti-rat 31470) or Cell Signaling Technology (goat anti-rat, 7077 S). Antibodies for loading controls were from Santa Cruz Biotechnology (anti-β-actin, sc-1615; anti-GAPDH, sc-20357) or Sigma-Aldrich (anti-β-tubulin, T8328). Fluorescent labelling for immunofluorescence was done with Alexa secondary antibodies (Thermo Fisher Scientific) using fluorophores with excitation at 488 (A-11017), 568 (A-11004) or 594 (A11072) at 1:1000 dilution.

### Co-immunoprecipitation (co-IP) and liquid chromatography-electrospray ionization-mass spectrometry (LC-ESI-MS)

Transfection of a monolayer of HEK293 cells in a 10 cm dish with 10 µg of V5-tagged SCHAD plasmid DNA was performed as described previously [6]. Crosslinking of cellular protein was carried out 48 h after transfection by a 10 min incubation with 1% paraformaldehyde in phosphate-buffered saline (PBS) at room temperature, followed by quenching with 1.25 M glycine/PBS and washing with PBS. All the subsequent steps were carried out at 4 ⁰C. Endogenous K8 was immunoprecipitated using the Pierce co-IP kit (Thermo Fisher Scientific, 26149), after the manufacturer’s instructions with the following specifications: After lysis of the cell monolayer, the supernatant was used for protein quantification to adjust the protein concentration and load the same protein amount per sample. The cell lysate was incubated overnight with agarose beads coupled to the M20 anti-K8 antibody. Control agarose resin from the kit served as negative control. The HPA039588 antibody was used to detect SCHAD protein after co-IP, whereas K8 was detected by the M20 antibody.

Two technical replicates from previously published SCHADKO HEK293 co-IP experiments [6] were submitted for LC-ESI-MS analysis at the proteomics unit (PROBE) at the University of Bergen, Bergen, Norway. Briefly, these samples were obtained by immunoprecipitation of either overexpressed V5-tagged SCHAD or a V5-tagged SCHAD mutant that lacked the mitochondrial import signal (Δ1–12 SCHAD-V5). Mouse IgG2a antibody (DAKO, X0943) was used for the negative controls. To prepare the samples for LC-ESI-MS, protein cleanup of the co-IP samples was performed with Sera-Mag beads (GE Healthcare) and digestion was done in-column with porcine trypsin (Promega). Following peptide extraction, desalting was done with OASIS C18 96-well plates. Samples were run on a Q Exactive HF Orbitrap coupled to an Ultimate 3000 Rapid Separation LC system (Thermo Fisher Scientific). Raw files were processed with MaxQuant software (Version 1.6.1.0) [22] and the Perseus 1.6.1.1 platform [23] was used to analyze and visualize protein groups.

### Western blotting

Protein samples were mixed with NuPAGE LDS sample buffer and reducing agent (Thermo Fisher Scientific, NP0007 and NP0004) and heated at 70 °C for 10 min before separation by SDS-PAGE and subsequent transfer to a PVDF membrane. Proteins were visualized by enhanced chemiluminescence using ECL Prime or Select Western Blotting detection reagents (GE Healthcare, RPN2232 and RPN2235). The membranes were imaged on a GBOX I Chemi XR5 imager (Syngene).

### Immunofluorescence

HEK293 cells were grown on poly-L-lysine-coated coverslips. They were transfected with V5-tagged SCHAD expression plasmid and fixed 48 h after transfection using 100% ice-cold methanol for 3 min. Immunostaining was performed as in [6]. EndoC-βH1cells were grown on coverslips coated by βCoat and fixed using 100% ice-cold methanol for 3 min at -20 °C. The cells were blocked with 5% goat serum in PBS for 30 min, followed by incubation with the GTX105167 anti-SCHAD antibody overnight in blocking solution. They were then washed for 3 × 10 min with PBS/Tween 0.05% (PBST), followed by incubation with anti-K8 (M20) for 1 h. The cells were washed 3 × 10 min in PBST and consecutively incubated with secondary antibodies (A11017 and A11072), each at 1:1000 dilution, for 1 h at room temperature. After a final 3 × 10 min wash in PBST, the nuclei were stained with DAPI and mounted in ProLong Glass Antifade media (Thermo Fisher Scientific). The images were acquired with a TCS SP8 STED 3X (Leica Microsystems) confocal microscope and a 100x/NA 1.4 HC PL APO STED WHITE objective with the appropriate laser lines and emission detection ranges. Z-stacks were acquired with a step size of 0.45 μm. The images were processed using adaptive deconvolution in the LasX Lightning module (Leica Software).

### Glucose-stimulated insulin secretion (GSIS) assay on EndoC-βH1 cells

EndoC-βH1 cells grown in 8-well chambers were incubated for 24 h in ULTI-ST glucose starvation medium (Human Cell Design) prior to GSIS to make sure that all cells were at basal rate. The medium was removed, and the cells were washed with βKrebs solution (Human Cell Design) supplemented with 0.1% BSA (Krebs-BSA) twice before the cells were incubated in Krebs-BSA for 1 h. The cells were then stimulated by adding Krebs-BSA containing 20 mM glucose, while control cells were in parallel incubated without glucose. Cells were fixed in ice-cold methanol 10, 30 and 40 min after the start of stimulation. All incubations were performed in an incubator at 37 °C with 5% CO_2_ and saturated humidity.

### Proximity ligation assay (PLA)

PLA [24] was performed on fixed EndoC-βH1 cells using the Duolink Proximity Ligation Assay (Merck, DUO92101). The coverslips were placed in a 6-well cell culturing plate during the staining protocol. Drawing with a hydrophobic ImmEdge pen (Vector Laboratories, USA) was used to keep the fluid in place, and all incubations were conducted in a humidity chamber. Buffers and solutions were prepared according to the DuoLink PLA protocol. Following 1 h of blocking at 37 °C with one drop of Duolink Blocking Solution, endogenous SCHAD protein was labelled using the GTX105167 antibody diluted 1:1000 in Duolink Antibody Diluent (40 µl per coverslip) and incubated at 4 °C overnight. Next morning, the coverslips were washed 2 × 10 min on a shaker using Wash Buffer A and endogenous K8 labelled using the M20 antibody diluted 1:1000 (40 µl per coverslip), followed by incubation at room temperature for 1 h. Negative controls were GTX105167 (overnight incubation) combined with IgG mouse isotype control (Invitrogen, 10400 C) for 1 h, and M20 (1 h) combined with IgG rabbit isotype control (Invitrogen, 10500 C) overnight, in addition to omitting the primary antibodies (‘probe only’).

The coverslips were washed as above, and PLUS and MINUS PLA probes (diluted 1:5 in Antibody diluent, 40 µl per coverslip) were added. The coverslips were incubated at 37 °C for 1 h, washed 2 × 10 min with Wash Buffer A on a shaker and 40 µl of Duolink Ligase (diluted 1:40 in 1x Ligation Buffer) added, followed by incubation for 30 min at 37 °C. The coverslips were washed 2 × 5 min in Wash Buffer A on a shaker. During the last wash cycle, the polymerase was diluted 1:80 in 1x Amplification Buffer (Red) in darkness and 40 µl was added, followed by a 100 min incubation at 37°C. From this point on the coverslips were kept in the dark. They were washed 2 × 20 min in 1x Wash Buffer B on a shaker, then for 1 min in 0.01x Wash Buffer B and dried at room temperature. The coverslips were mounted on a glass slide using 7 µl of Duolink PLA Mounting Medium and edges sealed with nail polish. The slides were stored at 4°C and analyzed the next day.

Confocal microscopy (TCS SP8 STED 3X, Leica Microsystems) was used for image acquisition. Images were taken using a 40x/NA 1.1 HC PL APO motCORR CS2 water immersion objective using identical lasers settings for all samples within each experiment. The number of PLA signals were quantified using the AggreCount macro [25] in the Fiji software [26]. A region of interest (ROI) was drawn around a small subset of cells and the number of PLA signals and nuclei (= cells) were counted. For each sample, 8–10 ROIs were analyzed, each containing 5–8 cells. Results are given as average number of PLA signals/cell.

### Experimental animals and islet isolation

The full body SCHAD knockout mouse strain (SCHADKO) was maintained on a mixed genetic background (B6/129) and genotyped as described [8]. These animal studies were approved by the Institutional Review Board of Joslin Diabetes Center and were in accordance with National Institutes of Health (NIH) guidelines. The animals were kept under a 12-h light cycle and fed ad libitum. They were euthanized by asphyxiation with CO_2_ followed by cervical dislocation. Full body K8 knockout (K8KO) mice and controls on an FVB/n background were generated through breeding of heterozygous animals and genotyped as previously described [27]. Samples from K8KO mice and littermate controls including the ketogenic samples were obtained via previous studies [15, 28]. Thus, no new K8KO animals were bred for the purpose of the present study. Pancreatic tissue was collected immediately after euthanasia and islets from SCHADKO and K8KO mice and controls were isolated according to previously described protocols [15, 29]. Freshly isolated islets were hand-sorted under a microscope on ice for lysate preparation.

### Data processing and visualization

Graphs and statistical analysis were done using GraphPad Prism 7 (GraphPad Software, Boston). For Western blot quantification a nonparametric t-test was used. Two way-ANOVA followed by multiple unpaired t-tests was performed on the PLA quantifications. Data are shown as mean ± SEM. Protein structures and interactions were predicted by AlphaFold 3 [30, 31] and visualized using ChimeraX-1.9 [32]. The predicted alignment error (PAE) plot was visualized using PAE viewer [33]. The JSON files and other files from the AlphaFold 3 modelling are included as a Supplementary Folder.

## Results

### Identification of SCHAD interacting partners by Y2H screening

**Fig. 1:**
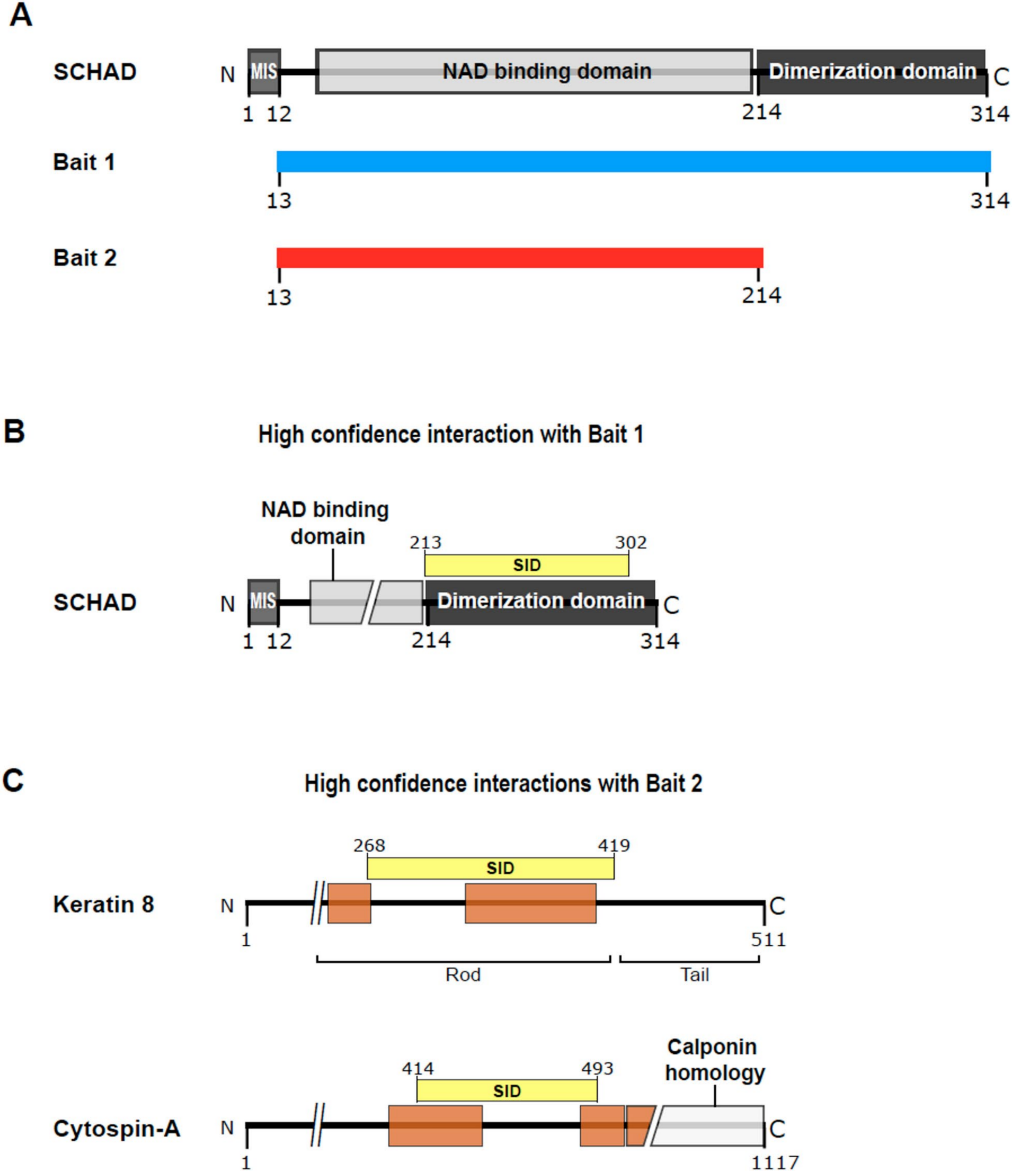
A putative SCHAD-K8 interaction identified by yeast 2-hybrid (Y2H) screening. (**A**) Schematic representation of the SCHAD protein and its domains, and the SCHAD protein variants used as bait in the two Y2H screens. Bait 1 lacked the mitochondrial import signal (MIS), while Bait 2 lacked the MIS and the dimerization domain. (**B**) Bait 1 resulted in the SCHAD protein itself being identified as the only prey with a high predicted biological score (PBSc). (**C**) Bait 2 resulted in two preys with high PBSc: K8 and cytospin-A. Legend: Brown boxes = coiled-coil domains; yellow boxes = the specific interaction domains (SID) identified by the experiment; numbers = amino acid residue number. Numbers for K8 are according to its long isoform (P05787-2)

To search for SCHAD interaction partners that could be particularly relevant for its role in regulating insulin secretion, two Y2H screens were performed using a cDNA library constructed from human islets. The first screen was carried out using the full-length SCHAD protein (without the mitochondrial import signal) as bait (Fig. 1A, Bait 1). For the second screen, the dimerization domain was deleted (Fig. 1A, Bait 2). With Bait 1, SCHAD itself was the only protein interaction identified with a very high PBSc (Fig. 1B; Suppl. Figure S1; Table 1). The specific interaction domain (SID) spanned SCHAD amino acid residues 213 to 302, which coincided with the dimerization domain. It is known that SCHAD homodimerization is necessary for its stability and activity [34], and the first screen therefore reflected the high affinity that SCHAD has for itself. Thus, although the initial Y2H screen did not reveal any novel high-confidence interactions it demonstrated that our methodological approach worked.

Once the dimerization domain was removed and a second screen performed, SCHAD was not among the captured interactions, as expected. Instead, we obtained two new interactions with very high PBSc: keratin 8 (K8; gene name: *KRT8*) and cytospin-A (CYTSA, gene name: *SPECC1L*) (Fig. 1C; Suppl. Figure S2; Table 1). Strikingly, 309 of the 352 sequenced positive colonies (88%) corresponded to K8 (long isoform 2, transcript ID NM_001256282.2, UniProt ID P05787-2) while only 19 (5%) corresponded to CYTSA. The SID spanned residues 268 to 419 for K8 and residues 414 to 493 for CYTSA, both containing α-helical domains (Fig. 1C).

**Table 1:** Top-ranked preys found in the Y2H screens using SCHAD baits 1 and 2. The predicted biological score (PBSc) ranks the reliability of each interaction in six categories: A, very high confidence; B, high confidence; C, good confidence; D, moderate confidence where there can be false-positive interactions or interactions hardly detectable by the technique; E, highly connected preys that do not display specific interactions; and F, experimentally proven technical artefacts. Frequency shows the number of identified clones for each protein among all identified clones in the respective Y2H screens

	Gene ID	Gene symbol	Protein name	Protein symbol	PBSc	Frequency
**Bait 1**	3033	*HADH*	Short-chain 3-hydroxyacyl-CoA dehydrogenase	SCHAD	A	405/441
	10114	*HIPK3*	Homeodomain interacting protein kinase 3	HIPK3	D	1/441
	4709	*NDUFB3*	NADH dehydrogenase [ubiquinone] 1 beta subcomplex subunit B3	NDUFB3	D	2/441
	10210	*TOPORS*	TOP1 binding arginine/serine-rich protein	TOPORS	D	1/441
	7750	*ZMYM2*	Zinc finger MYM-type containing 2	ZMYM2	D	1/441
**Bait 2**	3856	*KRT8*	Keratin, type II cytoskeletal 8	K8	A	309/352
	23384	*SPECC1L*	Sperm antigen with calponin homology and coiled-coil domains 1 like	CYTSA	A	19/352
	64283	*ARHGEF28*	Rho guanine nucleotide exchange factor 28	ARG28	D	1/352
	66005	*CHID1*	Chitinase domain containing 1	SICLP	D	3/352
	57572	*DOCK6*	Dedicator of cytokinesis 6	DOCK6	D	1/352
	8541	*PPFIA3*	PTPRF interacting protein alpha 3	LIPA3	D	2/352

### Evaluation of the K8-SCHAD interaction in HEK293 cells

**Fig. 2:**
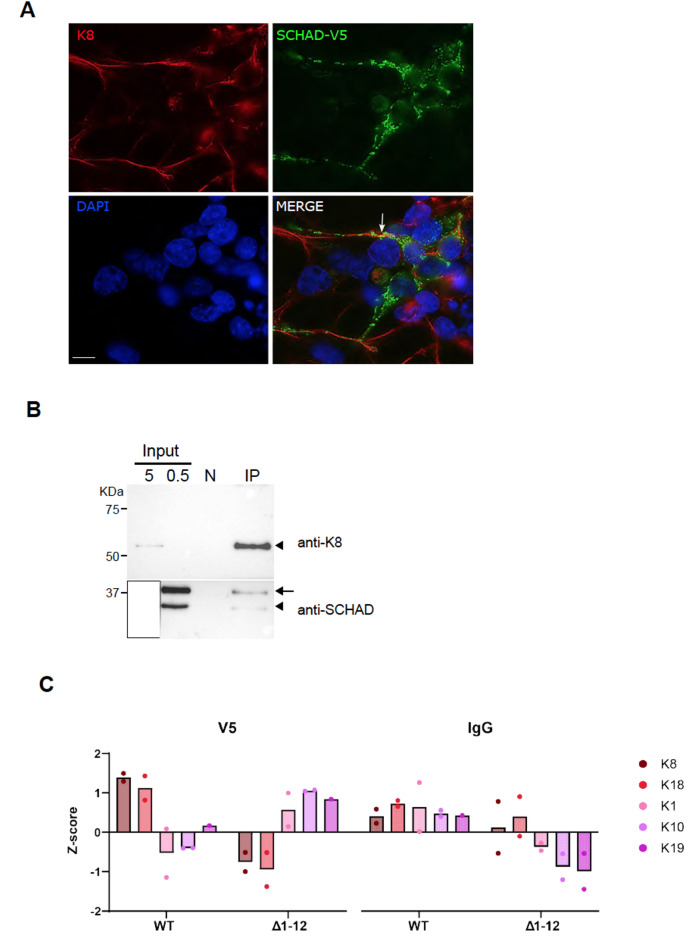
Subcellular localization and co-immunoprecipitation (co-IP) of SCHAD and K8 from HEK293 cells. (**A**) HEK293 cells transfected with V5-tagged SCHAD were fixed and stained with anti-V5 (green) and anti-K8 (red) antibodies. Counterstaining of nuclei was with DAPI (blue). Imaging was done by regular fluorescence microscopy. The arrow points to an area of yellow signal, indicating co-localization. Scale bar = 10 μm. (**B**) HEK293 cells were transfected with V5-tagged SCHAD and crosslinked with 1% paraformaldehyde. The cell lysate was immunoprecipitated with the anti-K8 M20 antibody, followed by western blotting of the precipitate. The upper part of the membrane was immunostained for K8 and the lower part for SCHAD. The arrowheads indicate endogenous K8 and SCHAD, whereas the arrow points to tagged SCHAD protein resulting from the transfection. Two different amounts of cell lysate were loaded on the gel (0.5 and 5 µg protein) to visualize the weak K8 band in the input of the experiment. The strong SCHAD signal in the 5-µg lane had to be covered (white box) to avoid overexposure. Negative control (N) was control agarose resin. The IP lane shows the signals in the immunoprecipitate. The experiment was repeated 3 times with similar results. (**C**) HEK293 cells without endogenous SCHAD protein (SCHADKO) were transfected with normal SCHAD-V5 (WT) or a mutant SCHAD-V5 variant without a mitochondrial import signal (Δ1–12). After crosslinking, the cell lysates underwent co-IP by either an anti-V5 antibody or a general anti-IgG antibody. Finally, the immunoprecipitates were subjected to mass spectrometry. Two technical replicates were analyzed. The figure shows the Z-score for keratins identified in HEK293 cells (K1, K8, K10, K18, K19)

K8 is the main type II keratin of the endocrine pancreas, with implications for islet and β-cell structural integrity, mitochondrial morphology and insulin secretion when the protein is missing [14–16]. We therefore decided to explore whether interaction between SCHAD and K8 does occur in human cell types. For validation, we first employed the HEK293 cell line, which has been established as a cellular model for studying many functional aspects of SCHAD [6]. Since these cells express SCHAD at relatively low levels, we initially overexpressed the protein fused with a V5 tag to enhance detectability. By immunofluorescence (Fig. 2A), we confirmed the subcellular localizations of K8 and SCHAD: K8 forms filamentous, cytoplasmic heteropolymers with type I keratins in some simple epithelial tissues [13, 35, 36], whereas SCHAD is a ubiquitously expressed enzyme effectively transported to the mitochondria due to its mitochondrial import signal [6]. Although we noted that the SCHAD-positive mitochondria tended to co-localize with the intermediate filaments formed by K8, only in a few areas of the HEK293 cells did we observe yellow signals that could indicate that the two proteins were positioned closely together (Fig. 2A).

We proceeded to test the putative interaction by co-IP, employing lysates from the HEK293 cells transiently transfected with SCHAD-V5. The cells were treated with a cross-linking reagent that stabilized protein-protein interactions, followed by capture of K8 from the lysate using the M20 anti-K8 antibody. This resulted in co-IP of the overexpressed as well as the endogenous SCHAD protein (Fig. 2B).

In a follow-up experiment, we used HEK293 cells with disrupted *HADH* gene (SCHADKO HEK293, see [6]) to prevent competition of overexpressed SCHAD-V5 with endogenous SCHAD when further assessing the binding to K8. To test whether the intracellular localization of SCHAD influenced the interaction, we overexpressed either the normal SCHAD (WT SCHAD-V5) or a mutant SCHAD-V5 variant lacking the mitochondrial import signal (Δ1–12 SCHAD-V5) in the SCHADKO HEK293 cells.

The latter protein variant was unable to enter the mitochondria and instead remained in the cytoplasm (Suppl. Figure S3). We employed the anti-V5 antibody to capture over-expressed WT SCHAD-V5 or Δ1–12 SCHAD-V5 protein, or a general IgG antibody to serve as negative control for unspecific keratin binding and epidermal keratin contaminants. The co-IP fractions were then analyzed by MS. We checked the list of identified proteins captured by WT SCHAD and noted that GDH, the only well-established interactor with SCHAD [8], was among the top 25 hits (Suppl. Table S1). This observation supported the validity of the experiment, and we then specifically assessed the levels of keratins present in HEK293 cells (K1, K8, K10, K18, K19) (Fig. 2C). K8 and its partner K18, both non-epidermal keratins, were most abundant (Z-scores, i.e. standard deviations from the mean, of 1.7 and 1.5, respectively) in the samples where normal SCHAD had been immunoprecipitated and appeared different from the negative control. Notably, the Δ1–12 SCHAD-V5 sample contained the lowest amount of K8 and K18 in the experiment (Fig. 2C). This might be surprising due to the cytosolic localization of both this mutant variant of SCHAD and the keratins. The other three identified keratin molecules displayed a more homogenous pattern of abundance across all samples, making an interaction with SCHAD less likely (Fig. 2C).

### SCHAD and K8 interact during glucose-stimulated insulin secretion in a human β-cell line

**Fig. 3:**
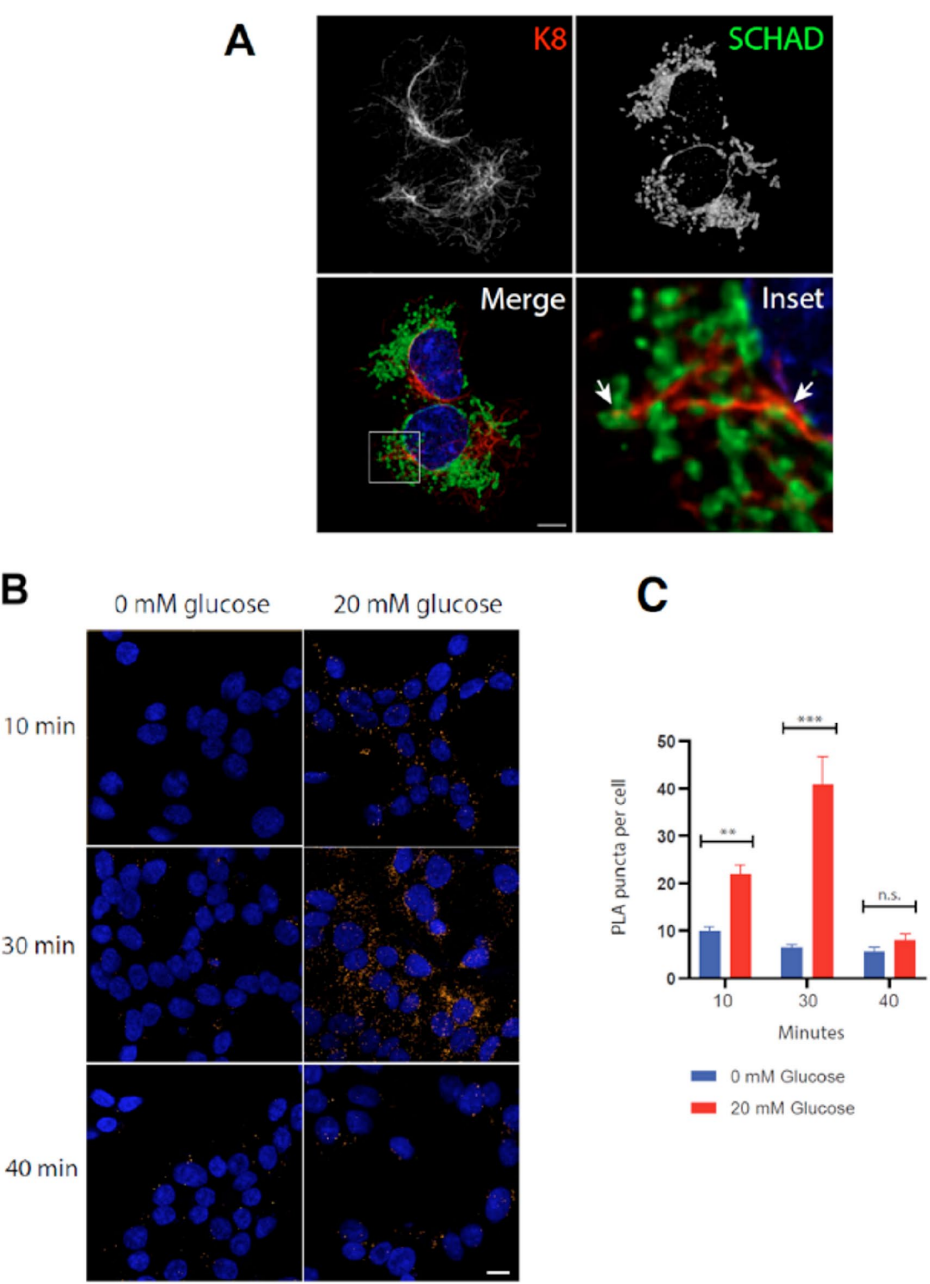
SCHAD and K8 interact during insulin secretion in the human β-cell line EndoC-βH1. **(A**) Immunofluorescent staining of EndoC-βH1 cells using antibodies against SCHAD and K8. The separate images of K8 and SCHAD are maximum projections of several planes through the cells, while the merged image and inset are single planes. Signals from K8 (green) and SCHAD (red) are in close proximity at some spots (arrows). Scale bar = 5 μm. (**B**) Proximity ligation assay (PLA) of EndoC-βH1cells undergoing glucose-stimulated insulin secretion. The cells were fixed 10, 30 or 40 min after addition of 20 mM glucose. Cells kept at 0 mM glucose were fixed at the same time points for reference. PLA was performed using antibodies against SCHAD and K8. Each orange dot represents a co-localization signal. (**C**) Quantification of the PLA signals in B. Data are represented as mean ± SEM. ** indicates *p* < 0.01, *** indicates *p* < 0.001, n.s. = not significant. A total of 300 cells were analyzed

Taken together, the co-IP experiments of Fig. 2 supported that SCHAD could be interacting with K8 in HEK293 cells. Because these cells originate from embryonic kidney, we pursued the investigation of a relationship between SCHAD and K8 in the human β-cell model EndoC-βH1, a cell line relevant for insulin secretion [21]. Immunofluorescent double-staining followed by confocal microscopy confirmed that EndoC-βH1 cells express K8 and SCHAD, and that these proteins have the expected subcellular locations (Fig. 3A). The K8 filaments and mitochondria were often positioned closely together, but only in a few instances did the staining suggest intimate co-localization of K8 and SCHAD (inset of Fig. 3A). Given the limitations of the imaging method, it was not possible to determine whether the overlapping staining in EndoC-βH1 cells was due to K8 interacting with the outside of mitochondria or with the SCHAD protein in the mitochondrial matrix. We therefore decided to use proximity ligation assay (PLA), a method that can reveal protein-protein interactions via two secondary antibodies that are attached to DNA probes. Each secondary antibody binds to a primary antibody specific for one of the two interaction partners, and if the DNA probes are located within 40 nm of each other, they are ligated together and detected by a fluorescent signal [37].

The PLA experiment resulted in an average of around 10 puncta per EndoC-βH1 cell when grown in the OPTIβ culture medium that contains 5 mM glucose. The negative control reactions consistently displayed two or less puncta per cell (Suppl. Figure S4). We therefore concluded that there is a low, but statistically significant level of interaction between SCHAD and K8 in EndoC-βH1 cells growing under “steady state” conditions. Because these cells effectively secrete insulin when stimulated by high glucose [38], we examined whether the interaction between SCHAD and K8 changed during insulin secretion. A glucose-stimulated insulin secretion (GSIS) assay was performed by exposing starved EndoC-βH1 cells to high glucose (20 mM), followed by fixation at various time points. The PLA images were compared with data from parallel cells incubated without glucose (Fig. 3B). Quantification of the PLA puncta showed that after 10 min of GSIS, the number of SCHAD-K8 interactions had become significantly higher in the glucose-exposed cells, compared to the cells without glucose (22.1 ± 1.9 vs. 10.0 ± 0.9, *p* < 0.01). After 30 min the difference between the cells had increased further (40.8 ± 5.9 vs. 6.5 ± 0.7, *p* < 0.001 (Fig. 3C). However, at 40 min, when EndoC-βH1 cells incubated in high glucose return to baseline insulin secretion [38], there was no longer a significant difference between the two treatment groups.

### Expression of SCHAD and K8 in pancreatic tissue of knockout mouse models

**Fig. 4:**
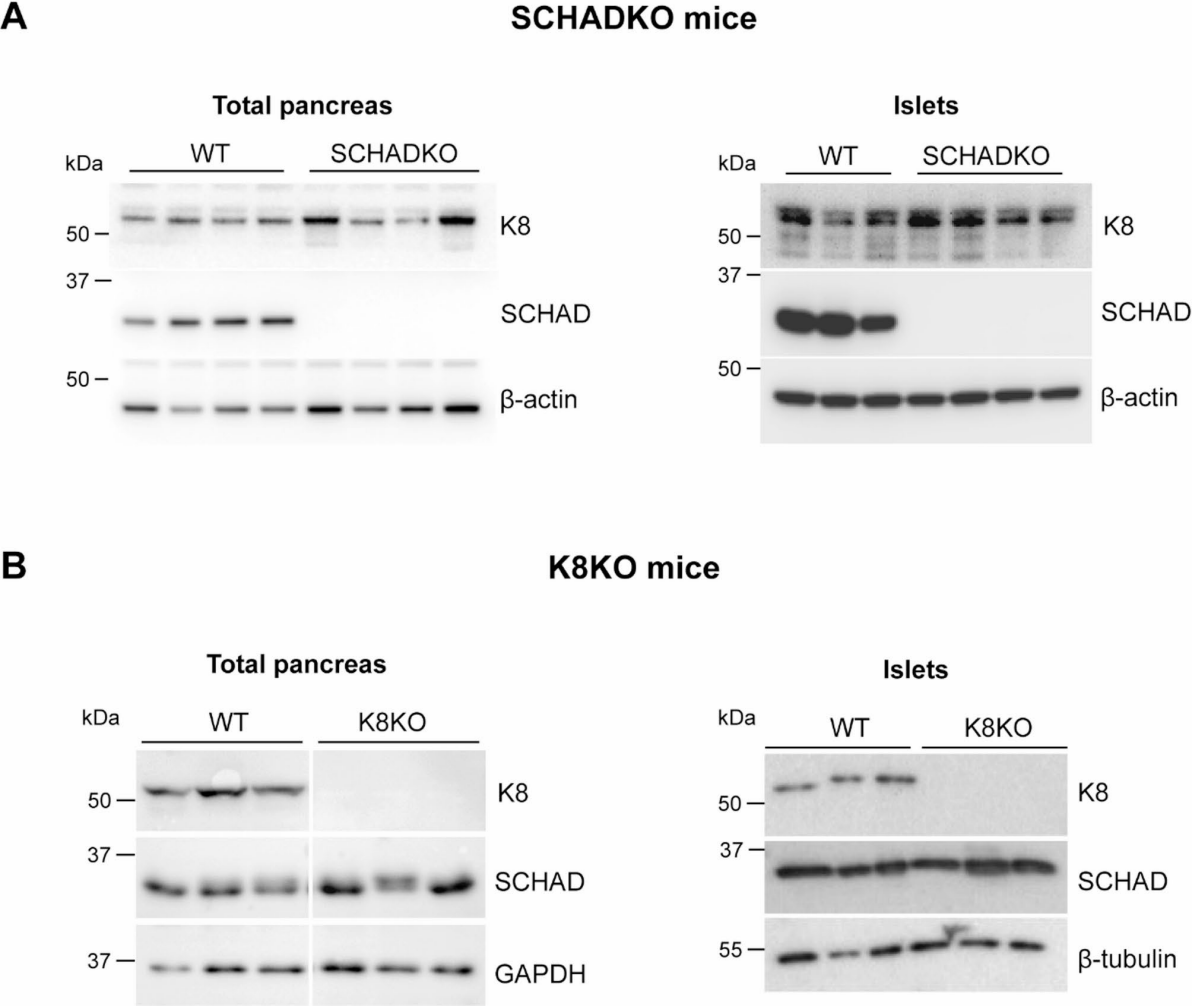
K8 and SCHAD expression in SCHAD and K8 knockout mice. (**A**) Western blots for the detection of K8 in lysates of total pancreas and isolated islets for WT and SCHADKO mice (*n* = 3 or 4). SCHAD protein was analyzed as confirmation of the knockout in SCHADKO samples and β-actin served as loading control. (**B**) Western blots for the detection of SCHAD in whole pancreas and isolated islets of WT and K8KO mice (*n* = 3). Knockout of K8 was confirmed by detection of K8, and GAPDH or β-tubulin served as loading control

If the SCHAD-K8 interaction observed in EndoC-βH1cells is of relevance for islet function in vivo, expression levels of the two proteins could influence each other. To test this, we evaluated pancreatic K8 expression in the SCHADKO mouse and SCHAD expression in the K8KO model. Lysates were examined both from the whole pancreas and isolated islets. However, protein abundance in the knockout lysates appeared very similar to the signals in lysates obtained from wildtype littermate mice (Fig. 4). Quantification of the bands upon adjusting to loading controls confirmed that there was no significant difference between knockout and wildtype animals (not shown). Thus, pancreatic K8 expression appears normal in SCHADKO animals and SCHAD expression normal in K8 knockouts.

**Fig. 5:**
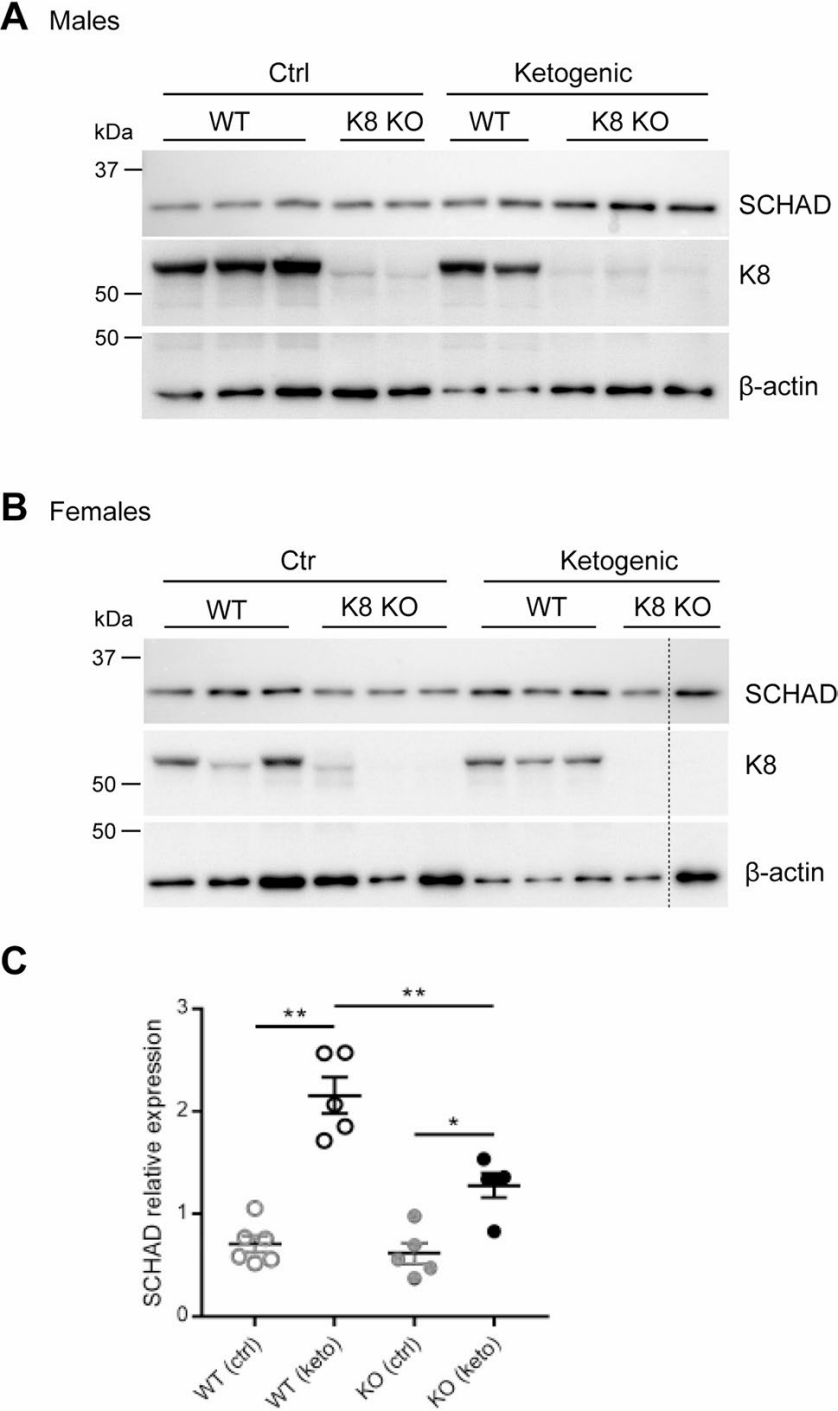
Pancreatic expression of SCHAD in K8 knockout mice fed a ketogenic diet. (**A**,** B**) Western blots for the detection of SCHAD in lysates of pancreas samples from male and female K8 wildtype (WT) (*n* = 11) and K8KO (*n* = 10) mice fed a control or ketogenic diet. K8 was detected for the confirmation of the wildtype or knockout genotype. Detection of β-actin served as loading control. The stippled line in B indicates a technically unsuccessful lane that was removed. (**C**) Quantification of SCHAD protein levels of (A) and (B) combined, normalized to respective β-actin loading controls. Data are represented as mean ± SEM. * indicates *p* < 0.05, ** indicates *p* < 0.01

K8 has been shown to modulate cell stress responses in the endocrine and exocrine pancreas. It is upregulated in β-cells after streptozotocin treatment as well as in β-cells derived from diabetic NOD mice [14]. To test whether an interaction between SCHAD and K8 could be relevant in a cell stress situation, we analyzed SCHAD expression in pancreas preparations of wildtype and K8KO mice fed either a control or ketogenic diet (Fig. 5A, B). The latter diet enhances fatty acid β-oxidation, in which SCHAD catalyzes the third step. SCHAD protein levels increased significantly when wildtype and K8KO mice were subjected to a ketogenic diet (wildtype control vs. ketogenic diet: 0.7 ± 0.08 vs. 2.2 ± 0.2, *p* = 0.004; K8KO control vs. ketogenic diet: 0.6 ± 0.1 vs. 1.3 ± 0.1, *p* = 0.02). However, this increase was blunted in the absence of K8 (ketogenic wildtype vs. ketogenic K8KO: 2.2 ± 0.2 vs. 1.3 ± 0.1, *p* = 0.008) (Fig. 5C).

### AlphaFold predicts an interaction between K8 and SCHAD

**Fig. 6:**
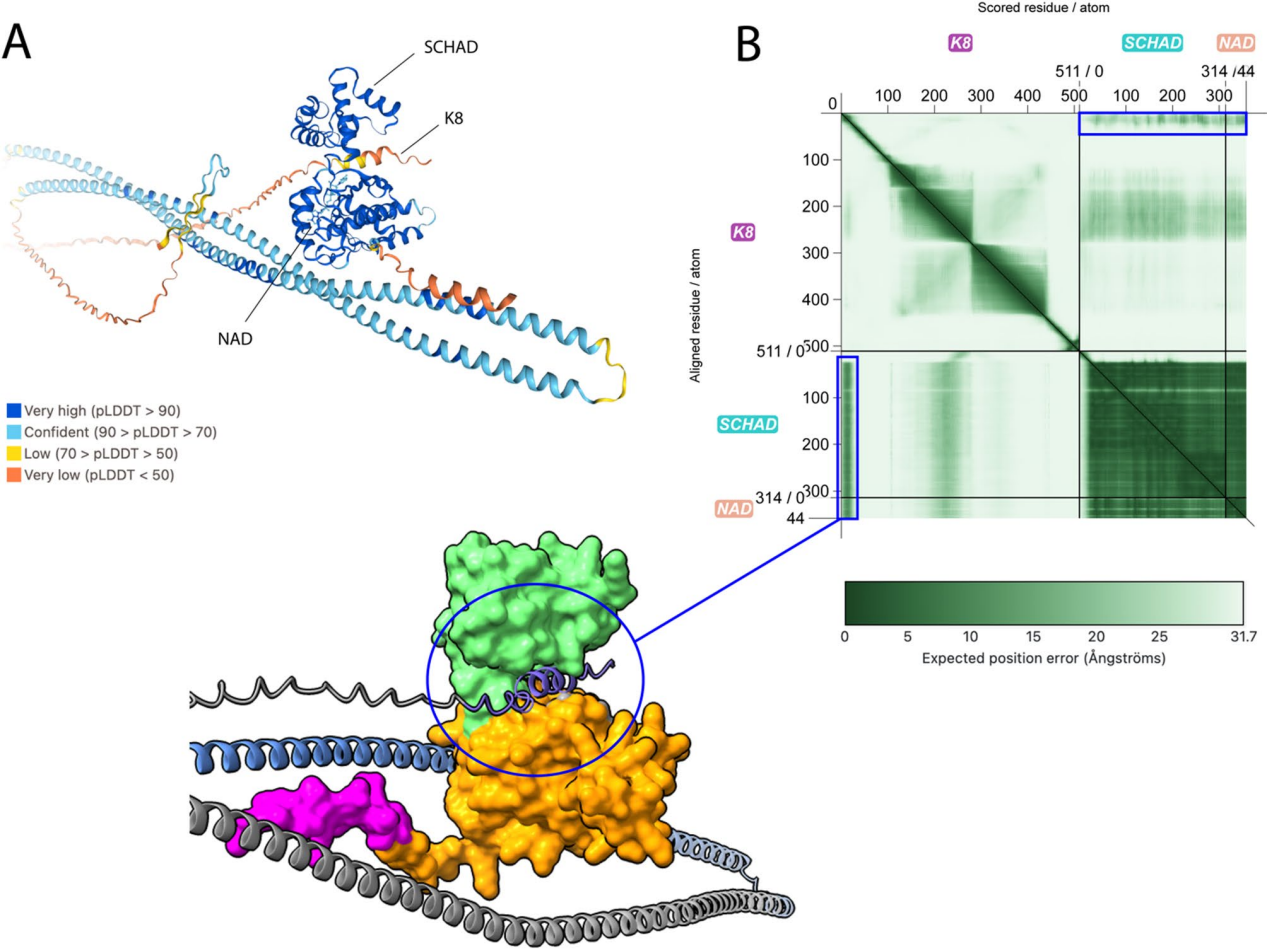
Structural modelling of a K8-SCHAD-NAD complex using AlphaFold 3. (**A**) A possible protein complex consisting of one K8 protein (UniProt ID P05787-2), one SCHAD protein (UniProt ID Q16836) and one NAD molecule is modelled and shown in colors representing the pLDDT score (an estimate of the confidence in the structure prediction). (**B**) The predicted alignment error (PAE) matrix shows a confident prediction of SCHAD while K8 has regions of both good and poor prediction, indicated by the intensity of the green color in the squares representing each protein (darker is more confident). The black lines indicate the chain boundaries. A region in the head of K8 (amino acids, AA 2–26), indicated by the blue boxes, shows a possible interaction with both SCHAD and NAD. The corresponding region of the protein complex is magnified in the blue circle. The SCHAD protein is shown with surfaces (Chimera-X) where pink represents the mitochondrial import signal, yellow the NAD-binding domain and green the dimerization domain. K8 is shown in purple (AA 2–26), blue (AA 268–419, corresponding to the Y2H specific interaction domain) and grey (other parts). The bound NAD is not visible in this surface model

To further evaluate the likelihood of a K8-SCHAD interaction, we used AlphaFold 3 [30, 31] to model the human K8 protein (isoform 2, P05787-2) along with one molecule of SCHAD (Q16836) bound to its cofactor nicotinamide adenine dinucleotide (NAD) (Fig. 6A). The crystal structure of SCHAD has previously been solved [39], and AlphaFold 3 predicts a structure with good correspondence to the true structure (chain predicted template modeling score (PTM): 0.81). On the other hand, the structure of K8 has not been experimentally determined. Also, this protein is predicted with disorganized regions in the head and tail, which results in a low chain-PTM score (0.23), and only its coiled-coil domains were modeled with relatively high confidence (Fig. 6A, B). The chain pair interface-predicted TM (iPTM) score for the protein complex was 0.27, which is very low [31]. Still, for proteins that contain regions not structurally well defined, the predicted alignment error (PAE) is a better parameter when evaluating interactions. Our PAE plot (Fig. 6B) shows a region where the N-terminus of K8 (head domain) could be interacting with SCHAD, indicated by a dark green square which indicates a low PAE value and therefore high confidence [31]. This region includes amino acids 2–26 of K8 and the main part of SCHAD. The NAD-binding and dimerization domains of the SCHAD protein form a cavity containing the active site, in which the head domain of K8 is proposed to bind (Fig. 6B). This model was stable between five different seed values, given that NAD was included in the analysis. The files resulting from the AlphaFold 3 prediction are provided in the Supplementary Folder.

## Discussion

The identification of protein-protein interactions is one basis for unravelling cellular signaling networks, understanding diseases that arise from their dysregulation, and pinpointing possible therapeutic targets. In the case of the genetic disorder CHI, it is known that the SCHAD protein serves to inhibit the enzyme GDH, and that hypoglycemia arises when this function fails [8]. Analysis of various tissues showed that SCHAD can interact with several other proteins than GDH [12]. The level of SCHAD expression is particularly high in the pancreatic β-cells [40] but partners of the protein have not been specifically explored in these cells. Since the identification of novel interactions could help clarify the role of SCHAD in insulin secretion, we performed Y2H screening of a cDNA library made from human islets. When the SCHAD protein without a dimerization domain was employed as bait, we revealed two unexpected, novel putative interacting partners: the epithelial intermediate filament protein K8 and the microtubule-associated protein cytospin-A.

Cytoskeletal proteins including intermediate filaments have in several cases been identified as interaction partners of soluble proteins by Y2H screening (see for example [41–44]). Hence, we chose to focus on K8 as this candidate clearly dominated the list of hits and had already been implicated in glucose homeostasis [14, 15, 45]. K8 together with K18 forms a cytoplasmic filamentous network that is important for structural integrity of the β-cell. Thus, specific knockout of K8 in mouse β-cells results in more fragile islets and disjointed plasma membrane organization with less β-cell expression of the Glut2 glucose transporter [16]. Moreover, the knockout leads to reduced GSIS in vivo although systemic glucose homeostasis appears normal [16].

Support for physical interaction between SCHAD and K8 was obtained by immunofluorescence, co-IP and PLA experiments. Nevertheless, it remains unclear how and where in the cell an interaction of SCHAD with K8 takes place. SCHAD and K8 are localized in different subcellular compartments as the former is targeted to the mitochondrial matrix while the latter forms part of the cytoplasmic cytoskeleton. Interestingly, a previous study identified multiple cytosolic enzymes including the glycolytic enzyme fructose-bisphosphate aldolase as interaction partners of SCHAD [12]. It is therefore possible that SCHAD interacts with cytosolic proteins including K8 prior to entering the mitochondrial matrix.

Nevertheless, we found that a SCHAD mutant lacking the mitochondrial import signal (Δ1–12), co-immunoprecipitated substantially less K8 and its partner K18 compared to normal SCHAD. One interpretation of this observation is that SCHAD must be inside the mitochondria to allow interacting with K8. If so, our finding suggests the possibility of a mitochondrial pool of K8 that has not been described to date. An intriguing hypothesis is that K8 serve as scaffold for assembly of the metabolic super-complexes with which SCHAD has been proposed to associate with [12], similar to the way that K8 forms a scaffold for the ERAD complex involved in protein degradation [46].

To further elucidate the postulated K8-SCHAD interaction, we used AlphaFold 3 [30, 31] to model the full-length K8 protein along with one molecule of SCHAD and the cofactor NAD (Fig. 6). The head region of K8 was predicted to associate with SCHAD, fitting inside the cavity between the NAD-binding domain and the dimerization domain of SCHAD where the active site of the enzyme is located [39]. Intriguingly, the first 28 amino acids in the N-terminus of K8 are present only in the long isoform of K8 (P05787-2). This raises the possibility that the long K8 isoform serves to keep SCHAD inactive until it reaches its final destination in the mitochondrial matrix. AlphaFold 3 was not able to predict an interaction between SCHAD and the SID of K8, as deduced from the Y2H screen. This could be due to uncertainties in the modelling or requirement of additional factors that are still unknown.

Notably, keratins and other intermediate filaments are emerging as important regulators of mitochondrial structure, function and intracellular distribution in different cell types, including β-cells [15, 47–50]. For example, K8 has been shown to interact with trichoplein-mitofusin complex on the mitochondrial outer membrane [51, 52] and the specific loss of K8 in β-cells causes decreased mitochondrial size and diffuse cristae [16]. Moreover, K8 has been shown to be partially externalized into the extracellular space by cancer cells, and this externalization is of biological significance in the tumor invasion process [53, 54]. Interestingly, the exposed domains of K8 were identified by antibody-binding epitopes on residues 353–367 (long isoform: 381–395) and 420–450 (long isoform: 448–478) [54], which partly overlap with the SID identified in our Y2H screening (residues 268–419, Fig. 1C). We speculate that some K8 molecules are internalized into the mitochondria by a mechanism analogous to K8 externalization in cancer cells. This would expose its interaction domain that could serve as an anchoring point for SCHAD.

Regardless of where the postulated K8-SCHAD interaction happens in the cell, its biological relevance is unclear. It is possible that this interaction occurs only in certain contexts, such as during cellular stress. In fact, K8 is upregulated in isolated islets stimulated with glucose [55] and also in β-cells of diabetic NOD mice and mice treated with streptozotocin [14]. In the human beta-cell line EndoC-βH1, we discovered that the number of SCHAD-K8 interactions, represented by PLA puncta, increased significantly during GSIS and reached a maximum after 30 min. As insulin secretion from EndoC-βH1 cell line peaks 10 min after glucose stimulation and then is reduced to basal secretion levels 30–40 min later [38], it is conceivable that during high demands for insulin, increased SCHAD mobilization to the mitochondria is involved in downregulating the secretory response. K8 might then potentially facilitate rapid transport of newly synthesized SCHAD to the mitochondria or stabilize the protein within the organelle. Alternatively, we speculate that K8 could scaffold SCHAD in the initial phase of glucose stimulation, i.e. when high insulin secretion is needed and the inhibitory effect of SCHAD needs to be restricted.

Our studies of K8 and SCHAD pancreatic expression levels in SCHADKO and K8KO mice, respectively, demonstrated that the absence of one protein did not affect the abundance of the other. Still, we noted that when K8KO mice were challenged with a ketogenic diet, upregulation of SCHAD was blunted in the pancreas of K8KO mice. Similarly, a previous study performed in K8KO mice fed a ketogenic diet, detected a blunted upregulation of mitochondrial 3-hydroxy-3-methylglutaryl-CoA synthase 2 (HMGCS2) in the colon, indicating impaired ketogenic metabolism [28]. This could suggest a role for K8 in adapting SCHAD expression to metabolic stress when there is an increased need for fatty acid oxidation in the mitochondria. However, it could also reflect a general state of mitochondrial dysfunction in K8KO animals.

Besides K8, the Y2H screen also identified cytospin-A as a possible interaction partner of SCHAD. Cytospin-A is a poorly characterized cytoskeletal cross-linking protein involved in actin cytoskeleton organization, microtubule stability and directional cell migration, and also associated with oblique facial clefting in humans [56]. Like K8, the SID of cytospin-A contains coiled-coil domains. Cytospin-A has not yet been implicated in β-cell function, and future studies should assess whether it is expressed in the pancreas and if so, whether an interaction with SCHAD is of any functional relevance for the β-cells.

Taken together, our results point to a possible interaction between SCHAD and K8, two proteins that are both implicated in β-cell biology. One limitation of the present work is that co-IP was performed in HEK293 cells and not in the human β-cell model or mouse islets. Moreover, species differences may play a role when interpreting these results. Besides K8, murine islets contain small amounts of K7 [14], a type II keratin that has not been reported for human islets. This may cause variations in how murine and human β-cells react to missing SCHAD protein.

For future investigations, we suggest that demonstration of a possible mitochondrial K8 pool should be attempted by super-resolution microscopy or other sensitive techniques. Additional studies should be undertaken in animal models, considering that general and β-cell-specific knockouts of both K8 and SCHAD are available [8, 9, 14, 16]. Notably, Toivola and coworkers [57] identified the SCHAD-interacting protein GDH as one of five mitochondrial autoantigens arising in K8-KO mice. An investigation of how downregulation or absence of GDH impacts the postulated SCHAD-K8 interaction will therefore be of particular interest.

## Conclusions

We propose that K8 serves to mobilize SCHAD to the mitochondria, to anchor/stabilize SCHAD within the organelle or to control its activity. Such functions could be of particular relevance when there is increased demand for this protein, e.g. under certain stress conditions. If so, an absence of the SCHAD-K8 interaction secondary to a defective SCHAD protein has the potential to influence the phenotype of CHI.

## Electronic supplementary material

Below is the link to the electronic supplementary material.

Supplementary Material 1

Supplementary Material 2

Supplementary Material 3

## Data Availability

All data generated or analysed during this study are included in this published article and its supplementary information files. Additional raw images of the PLA experiment and raw data from the quantifications are available from the corresponding author on reasonable request.

## References

[1] Rosenfeld E, Ganguly A, De Leon DD. Congenital hyperinsulinism disorders: genetic and clinical characteristics. Am J Med Genet Part C: Seminars Med Genet. 2019;181(4):682–92. 10.1002/ajmg.c.31737 PMC7229866 31414570

[2] Velde CD, Reigstad H, Tjora E, Guthe HJT, Hansen EV, Molven A, Njølstad PR. Congenital hyperinsulinism. Tidsskr nor Laegeforen 2023: 143(18). 10.4045/tidsskr.23.0425 38088279

[3] Maiorana A, Dionisi-Vici C. Hyperinsulinemic hypoglycemia: clinical, molecular and therapeutical novelties. J Inherit Metab Dis. 2017;40(4):531–42. 28656511 10.1007/s10545-017-0059-x

[4] Clayton PT, Eaton S, Aynsley-Green A, Edginton M, Hussain K, Krywawych S, Datta V, Malingré HEM, Berger R, van den Berg IET. Hyperinsulinism in short-chain L-3-hydroxyacyl-CoA dehydrogenase deficiency reveals the importance of β-oxidation in insulin secretion. J Clin Invest. 2001;108(3):457–65. 11489939 10.1172/JCI11294 PMC209352

[5] Molven A, Matre GE, Duran M, Wanders RJ, Rishaug U, Njølstad PR, Jellum E, Søvik O. Familial hyperinsulinemic hypoglycemia caused by a defect in the SCHAD enzyme of mitochondrial fatty acid oxidation. Diabetes. 2004;53(1):221–7. 14693719 10.2337/diabetes.53.1.221

[6] Velasco K, St-Louis JL, Hovland HN, Thompson N, Ottesen A, Choi MH, Pedersen L, Njølstad PR, Arnesen T, Fjeld K, et al. Functional evaluation of 16 SCHAD missense variants: only amino acid substitutions causing congenital hyperinsulinism of infancy lead to loss-of-function phenotypes in vitro. J Inherit Metab Dis. 2021;44(1):240–52. 32876354 10.1002/jimd.12309

[7] Molven A, Helgeland G, Sandal T, Njølstad PR. The Molecular Genetics and Pathophysiology of Congenital Hyperinsulinism Caused by Short-Chain 3-Hydroxyacyl-CoA Dehydrogenase Deficiency. In: Monogenic Hyperinsulinemic Hypoglycemia Disorders. 2012;21:137–145.

[8] Li C, Chen P, Palladino A, Narayan S, Russell LK, Sayed S, Xiong G, Chen J, Stokes D, Butt YM, et al. Mechanism of hyperinsulinism in short-chain 3-hydroxyacyl-CoA dehydrogenase deficiency involves activation of glutamate dehydrogenase. J Biol Chem. 2010;285(41):31806–18. 20670938 10.1074/jbc.M110.123638 PMC2951252

[9] St-Louis JL, El Jellas K, Velasco K, Slipp BA, Hu J, Helgeland G, Steine SJ, De Jesus DF, Kulkarni RN, Molven A. Deficiency of the metabolic enzyme SCHAD in pancreatic beta-cells promotes amino acid-sensitive hypoglycemia. J Biol Chem. 2023;299(8):104986. 37392854 10.1016/j.jbc.2023.104986 PMC10407745

[10] Filling C, Keller B, Hirschberg D, Marschall HU, Jörnvall H, Bennett MJ, Oppermann U. Role of short-chain hydroxyacyl coa dehydrogenases in SCHAD deficiency. Biochem Biophys Res Commun. 2008;368(1):6–11. 18036338 10.1016/j.bbrc.2007.10.188

[11] Stanley CA, Lieu YK, Hsu BYL, Burlina AB, Greenberg CR, Hopwood NJ, Perlman K, Rich BH, Zammarchi E, Poncz M. Hyperinsulinism and hyperammonemia in infants with regulatory mutations of the glutamate dehydrogenase gene. N Engl J Med. 1998;338(19):1352–7. 9571255 10.1056/NEJM199805073381904

[12] Narayan SB, Master SR, Sireci AN, Bierl C, Stanley PE, Li C, Stanley CA, Bennett MJ. Short-chain 3-hydroxyacyl-coenzyme a dehydrogenase associates with a protein super-complex integrating multiple metabolic pathways. PLoS ONE 2012: 7(4). 10.1371/journal.pone.0035048 PMC3322157 22496890

[13] Jacob JT, Coulombe PA, Kwan R, Omary MB. Types I and II keratin intermediate filaments. Cold Spring Harb Perspect Biol 2018: 10(4). 10.1101/cshperspect.a018275 PMC5880164 29610398

[14] Alam CM, Silvander JS, Daniel EN, Tao GZ, Kvarnstrom SM, Alam P, Omary MB, Hanninen A, Toivola DM. Keratin 8 modulates beta-cell stress responses and normoglycaemia. J Cell Sci. 2013;126(Pt 24):5635–44. 24144696 10.1242/jcs.132795 PMC3860309

[15] Silvander JSG, Kvarnstrom SM, Kumari-Ilieva A, Shrestha A, Alam CM, Toivola DM. Keratins regulate beta-cell mitochondrial morphology, motility, and homeostasis. FASEB J. 2017;31(10):4578–87. 28666985 10.1096/fj.201700095R

[16] Baghestani S, Haldin C, Kosijer P, Alam CM, Toivola DM. β-cell keratin 8 maintains islet mechanical integrity, mitochondrial ultrastructure and β-cell glucose transporter 2 plasma membrane targeting. Am J Physiol Cell Physiol. 2024;327(2):C462–C476. 10.1152/ajpcell.00123.2024 38912736

[17] Vojtek AB, Hollenberg SM. Ras-Raf interaction: two-hybrid analysis. Methods Enzymol. 1995;255:331–42. 8524119 10.1016/s0076-6879(95)55036-4

[18] Bartel P, Chien C-T, Sternglanz R, Fields S, Hartley DA. Using the two-hybrid system to detect protein-protein interactions. In: Hartley DA editor. Cellular interactions in development: a practical approach. Oxford University Press; 1993. p. 153–79.

[19] Fromont-Racine M, Rain JC, Legrain P. Toward a functional analysis of the yeast genome through exhaustive two-hybrid screens. Nat Genet. 1997;16(3):277–82. 9207794 10.1038/ng0797-277

[20] Formstecher E, Aresta S, Collura V, Hamburger A, Meil A, Trehin A, Reverdy C, Betin V, Maire S, Brun C, et al. Protein interaction mapping: a Drosophila case study. Genome Res. 2005;15(3):376–84. 15710747 10.1101/gr.2659105 PMC551564

[21] Ravassard P, Hazhouz Y, Pechberty S, Bricout-Neveu E, Armanet M, Czernichow P, Scharfmann R. A genetically engineered human pancreatic beta cell line exhibiting glucose-inducible insulin secretion. J Clin Invest. 2011;121(9):3589–97. 21865645 10.1172/JCI58447 PMC3163974

[22] Cox J, Mann M. MaxQuant enables high peptide identification rates, individualized p.p.b.-range mass accuracies and proteome-wide protein quantification. Nat Biotechnol. 2008;26(12):1367–72. 19029910 10.1038/nbt.1511

[23] Tyanova S, Temu T, Sinitcyn P, Carlson A, Hein MY, Geiger T, Mann M, Cox J. The perseus computational platform for comprehensive analysis of (prote)omics data. Nat Methods. 2016;13(9):731–40. 27348712 10.1038/nmeth.3901

[24] Alam MS. Proximity ligation assay (PLA). Curr Protoc Immunol. 2018;123(1):e58. 30238640 10.1002/cpim.58 PMC6205916

[25] Klickstein JA, Mukkavalli S, Raman M. AggreCount: an unbiased image analysis tool for identifying and quantifying cellular aggregates in a spatially defined manner. J Biol Chem. 2020;295(51):17672–83. 33454006 10.1074/jbc.RA120.015398 PMC7762942

[26] Schindelin J, Arganda-Carreras I, Frise E, Kaynig V, Longair M, Pietzsch T, Preibisch S, Rueden C, Saalfeld S, Schmid B, et al. Fiji: an open-source platform for biological-image analysis. Nat Methods. 2012;9(7):676–82. 22743772 10.1038/nmeth.2019 PMC3855844

[27] Baribault H, Penner J, Iozzo RV, Wilson-Heiner M. Colorectal hyperplasia and inflammation in keratin 8-deficient FVB/N mice. Genes Dev. 1994;8(24):2964–73. 7528156 10.1101/gad.8.24.2964

[28] Helenius TO, Misiorek JO, Nystrom JH, Fortelius LE, Habtezion A, Liao J, Asghar MN, Zhang H, Azhar S, Omary MB, et al. Keratin 8 absence down-regulates colonocyte HMGCS2 and modulates colonic ketogenesis and energy metabolism. Mol Biol Cell. 2015;26(12):2298–310. 25904331 10.1091/mbc.E14-02-0736 PMC4462946

[29] Gotoh M, Ohzato H, Dono K, Kawai M, Yamamoto H, Kanai T, et al. Successful islet isolation from preserved rat pancreas following pancreating ductal collagenase at the time of harvesting. Horm Metab Res Suppl.1990; 25:1–4. 1965177

[30] Jumper J, Evans R, Pritzel A, Green T, Figurnov M, Ronneberger O, Tunyasuvunakool K, Bates R, Žídek A, Potapenko A, et al. Highly accurate protein structure prediction with alphafold. Nature. 2021;596(7873):583–9. 34265844 10.1038/s41586-021-03819-2 PMC8371605

[31] Abramson J, Adler J, Dunger J, Evans R, Green T, Pritzel A, Ronneberger O, Willmore L, Ballard AJ, Bambrick J, et al. Accurate structure prediction of biomolecular interactions with alphafold 3. Nature. 2024;630(8016):493–500. 38718835 10.1038/s41586-024-07487-w PMC11168924

[32] Meng EC, Goddard TD, Pettersen EF, Couch GS, Pearson ZJ, Morris JH, Ferrin TE. UCSF chimerax: tools for structure Building and analysis. Protein Sci. 2023;32(11):e4792. 37774136 10.1002/pro.4792 PMC10588335

[33] Elfmann C, Stulke J. PAE viewer: a webserver for the interactive visualization of the predicted aligned error for multimer structure predictions and crosslinks. Nucleic Acids Res. 2023;51(W1):W404–10. 37140053 10.1093/nar/gkad350 PMC10320053

[34] Xu Y, Li H, Jin YH, Fan J, Sun F. Dimerization interface of 3-hydroxyacyl-CoA dehydrogenase tunes the formation of its catalytic intermediate. PLoS ONE. 2014;9(4):e95965. 24763278 10.1371/journal.pone.0095965 PMC3999109

[35] Ku NO, Zhou X, Toivola DM, Omary MB. The cytoskeleton of digestive epithelia in health and disease. Am J Physiol. 1999;277(6):G1108–1137. 10600809 10.1152/ajpgi.1999.277.6.G1108

[36] Omary MB, Ku NO, Strnad P, Hanada S. Toward unraveling the complexity of simple epithelial keratins in human disease. J Clin Invest. 2009;119(7):1794–805. 19587454 10.1172/JCI37762 PMC2701867

[37] Fredriksson S, Gullberg M, Jarvius J, Olsson C, Pietras K, Gustafsdottir SM, Ostman A, Landegren U. Protein detection using proximity-dependent DNA ligation assays. Nat Biotechnol. 2002;20(5):473–7. 11981560 10.1038/nbt0502-473

[38] Tsonkova VG, Sand FW, Wolf XA, Grunnet LG, Kirstine Ringgaard A, Ingvorsen C, Winkel L, Kalisz M, Dalgaard K, Bruun C, et al. The EndoC-βH1 cell line is a valid model of human beta cells and applicable for screenings to identify novel drug target candidates. Mol Metab. 2018;8:144–57. 29307512 10.1016/j.molmet.2017.12.007 PMC5985049

[39] Barycki JJ, O’Brien LK, Bratt JM, Zhang R, Sanishvili R, Strauss AW, Banaszak LJ. Biochemical characterization and crystal structure determination of human heart short chain L-3-hydroxyacyl-CoA dehydrogenase provide insights into catalytic mechanism. Biochemistry. 1999;38(18):5786–98. 10231530 10.1021/bi9829027

[40] Martens GA, Vervoort A, Van de Casteele M, Stange G, Hellemans K, Van Thi HV, Schuit F, Pipeleers D. Specificity in beta cell expression of L-3-hydroxyacyl-CoA dehydrogenase, short chain, and potential role in down-regulating insulin release. J Biol Chem. 2007;282(29):21134–44. 17491019 10.1074/jbc.M700083200

[41] Gentil BJ, McLean JR, Xiao S, Zhao B, Durham HD, Robertson J. A two-hybrid screen identifies an unconventional role for the intermediate filament peripherin in regulating the subcellular distribution of the SNAP25-interacting protein, SIP30. J Neurochem. 2014;131(5):588–601. 25113441 10.1111/jnc.12928

[42] Meng Y, Wu Z, Yin X, Zhao Y, Chen M, Si Y, Yang J, Fu X, Han W. Keratin 18 attenuates Estrogen receptor alpha-mediated signaling by sequestering LRP16 in cytoplasm. BMC Cell Biol. 2009;10:96. 20035625 10.1186/1471-2121-10-96 PMC2804594

[43] Oriolo AS, Wald FA, Canessa G, Salas PJ. GCP6 binds to intermediate filaments: a novel function of keratins in the organization of microtubules in epithelial cells. Mol Biol Cell. 2007;18(3):781–94. 17182859 10.1091/mbc.E06-03-0201 PMC1805110

[44] Vlachakis D, Tsilafakis K, Kostavasili I, Kossida S, Mavroidis M. Unraveling Desmin’s Head Domain Structure and Function. Cells 2024, 13(7). 10.3390/cells13070603 PMC11012097 38607042

[45] Roux A, Gilbert S, Loranger A, Marceau N. Impact of keratin intermediate filaments on insulin-mediated glucose metabolism regulation in the liver and disease association. FASEB J. 2016;30(2):491–502. 26467793 10.1096/fj.15-277905

[46] Pranke IM, Chevalier B, Premchandar A, Baatallah N, Tomaszewski KF, Bitam S, Tondelier D, Golec A, Stolk J, Lukacs GL, et al. Keratin 8 is a scaffolding and regulatory protein of ERAD complexes. Cell Mol Life Sci. 2022;79(9):503. 36045259 10.1007/s00018-022-04528-3 PMC11803007

[47] Steen K, Chen D, Wang F, Majumdar R, Chen S, Kumar S, Lombard DB, Weigert R, Zieman AG, Parent CA, et al. A role for keratins in supporting mitochondrial organization and function in skin keratinocytes. Mol Biol Cell. 2020;31(11):1103–11. 32213122 10.1091/mbc.E19-10-0565 PMC7353162

[48] Lowery J, Jain N, Kuczmarski ER, Mahammad S, Goldman A, Gelfand VI, Opal P, Goldman RD. Abnormal intermediate filament organization alters mitochondrial motility in giant axonal neuropathy fibroblasts. Mol Biol Cell. 2016;27(4):608–16. 26700320 10.1091/mbc.E15-09-0627 PMC4750921

[49] Schwarz N, Leube RE. Intermediate filaments as organizers of cellular space: how they affect mitochondrial structure and function. Cells 2016: 5(3). 10.3390/cells5030030 PMC5040972 27399781

[50] Nyström JH, Heikkilä TRH, Thapa K, Pulli I, Törnquist K, Toivola DM. Colonocyte keratins stabilize mitochondria and contribute to mitochondrial energy metabolism. Am J Physiol Gastrointest Liver Physiol. 2024;327(3):G438–53. 38860856 10.1152/ajpgi.00220.2023 PMC11427106

[51] Cerqua C, Anesti V, Pyakurel A, Liu D, Naon D, Wiche G, Baffa R, Dimmer KS, Scorrano L. Trichoplein/mitostatin regulates Endoplasmic reticulum-mitochondria juxtaposition. EMBO Rep. 2010;11(11):854–60. 20930847 10.1038/embor.2010.151 PMC2966954

[52] Nishizawa M, Izawa I, Inoko A, Hayashi Y, Nagata K, Yokoyama T, Usukura J, Inagaki M. Identification of trichoplein, a novel keratin filament-binding protein. J Cell Sci. 2005;118(Pt 5):1081–90. 15731013 10.1242/jcs.01667

[53] Weidle UH, Maisel D, Klostermann S, Schiller C, Weiss EH. Intracellular proteins displayed on the surface of tumor cells as targets for therapeutic intervention with antibody-related agents. Cancer Genomics Proteom. 2011;8(2):49–63. 21471515

[54] Albaret MA, Vermot-Desroches C, Pare A, Roca-Martinez JX, Malet L, Esseily J, Gerossier L, Briere J, Pion N, Marcel V et al. Externalized keratin 8: A target at the interface of microenvironment and intracellular signaling in colorectal Cancer cells. Cancers (Basel) 2018: 10(11). 10.3390/cancers10110452 PMC6266717 30453567

[55] Ahmed M, Bergsten P. Glucose-induced changes of multiple mouse islet proteins analysed by two-dimensional gel electrophoresis and mass spectrometry. Diabetologia. 2005;48(3):477–85. 15729580 10.1007/s00125-004-1661-7

[56] Saadi I, Alkuraya FS, Gisselbrecht SS, Goessling W, Cavallesco R, Turbe-Doan A, Petrin AL, Harris J, Siddiqui U, Grix AW, editors. Jr.: Deficiency of the cytoskeletal protein SPECC1L leads to oblique facial clefting. Am J Hum Genet 2011: 89(1):44–55. 10.1016/j.ajhg.2011.05.023 PMC3135813 21703590

[57] Toivola DM, Habtezion A, Misiorek JO, Zhang L, Nystrom JH, Sharpe O, Robinson WH, Kwan R, Omary MB. Absence of keratin 8 or 18 promotes antimitochondrial autoantibody formation in aging male mice. FASEB J. 2015;29(12):5081–9. 26399787 10.1096/fj.14-269795 PMC4653051

