# Supplementary material for: Searching for protein partners of short-chain 3-hydroxyacyl-CoA dehydrogenase (SCHAD) reveals keratin 8 as a novel candidate for interaction in pancreatic β-cells
Source: BMC Mol Cell Biol. 2025 Jun 5;26:18. doi: 10.1186/s12860-025-00544-w (PMC12139081; doi:10.1186/s12860-025-00544-w)

Supplementary Figure S1

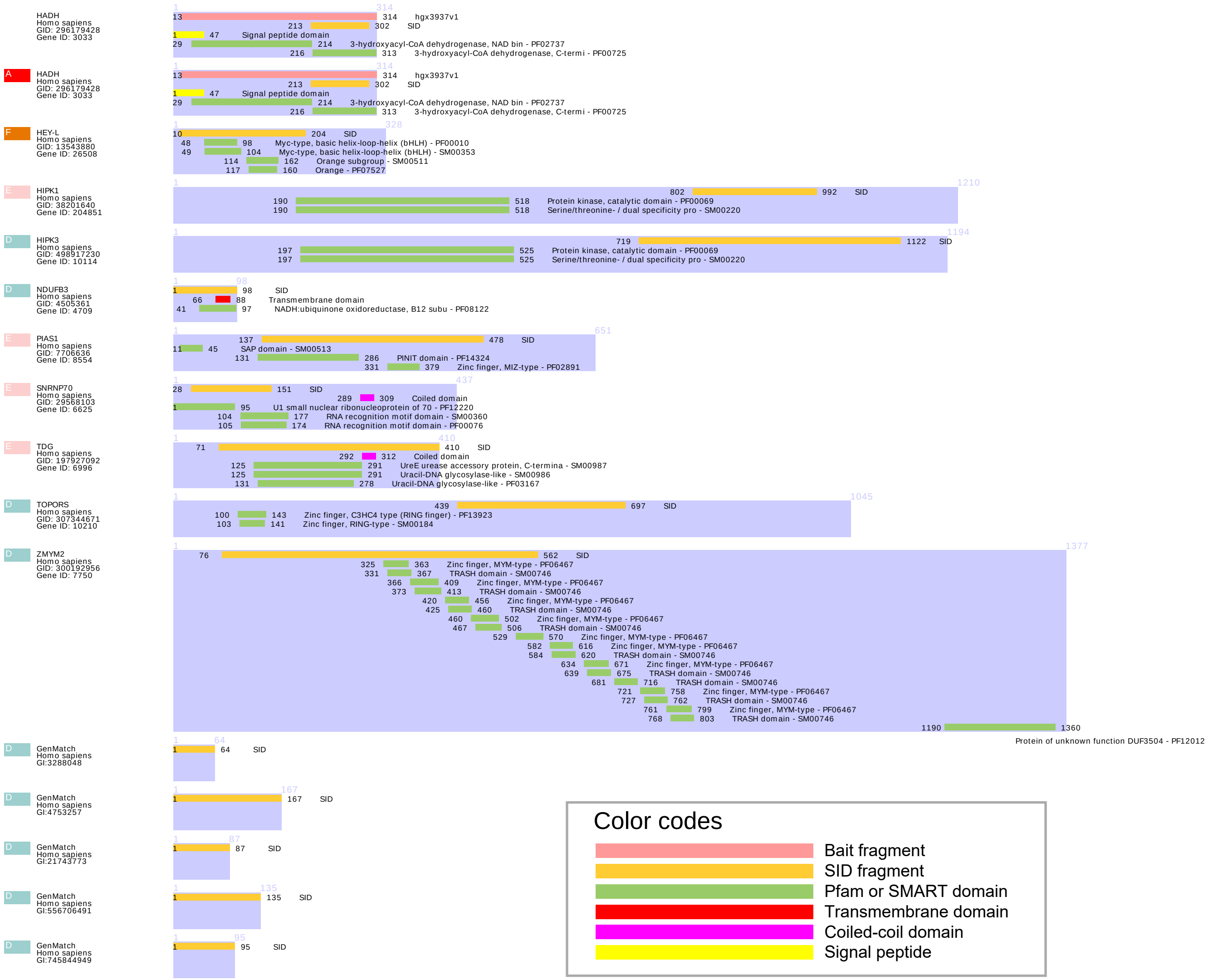

Supplementary Figure S1. Raw data from the Y2H screening with Bait 1 displaying all identified proteins. Bait 1 consisted of human *HADH* gene sequence coding for SCHAD amino acids 13-314. The bait and SID (selected interaction domain) fragments as well as domains of the identified proteins are color-coded as shown in the panel. The figure was provided by Hybrigenics Services.

Supplementary Figure S2

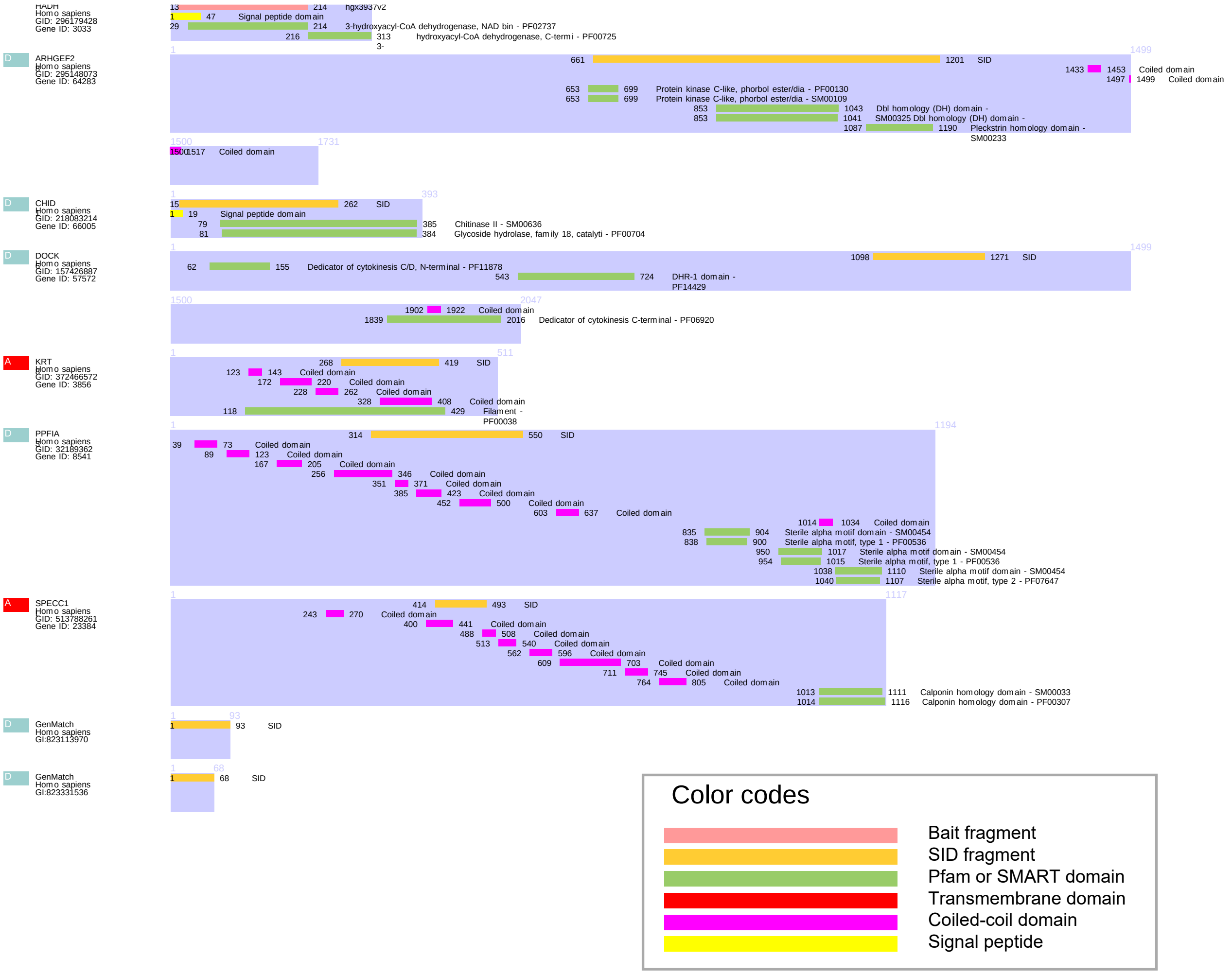

Supplementary Figure S2. Raw data from the Y2H screening with Bait 2 displaying all identified proteins. Bait 2 consisted of human *HADH* gene sequence coding for SCHAD amino acids 13-214. The bait and SID (selected interaction domain) fragments as well as domains of the identified proteins are color-coded as shown in the panel. The figure was provided by Hybrigenics Services.

Supplementary Figure S3

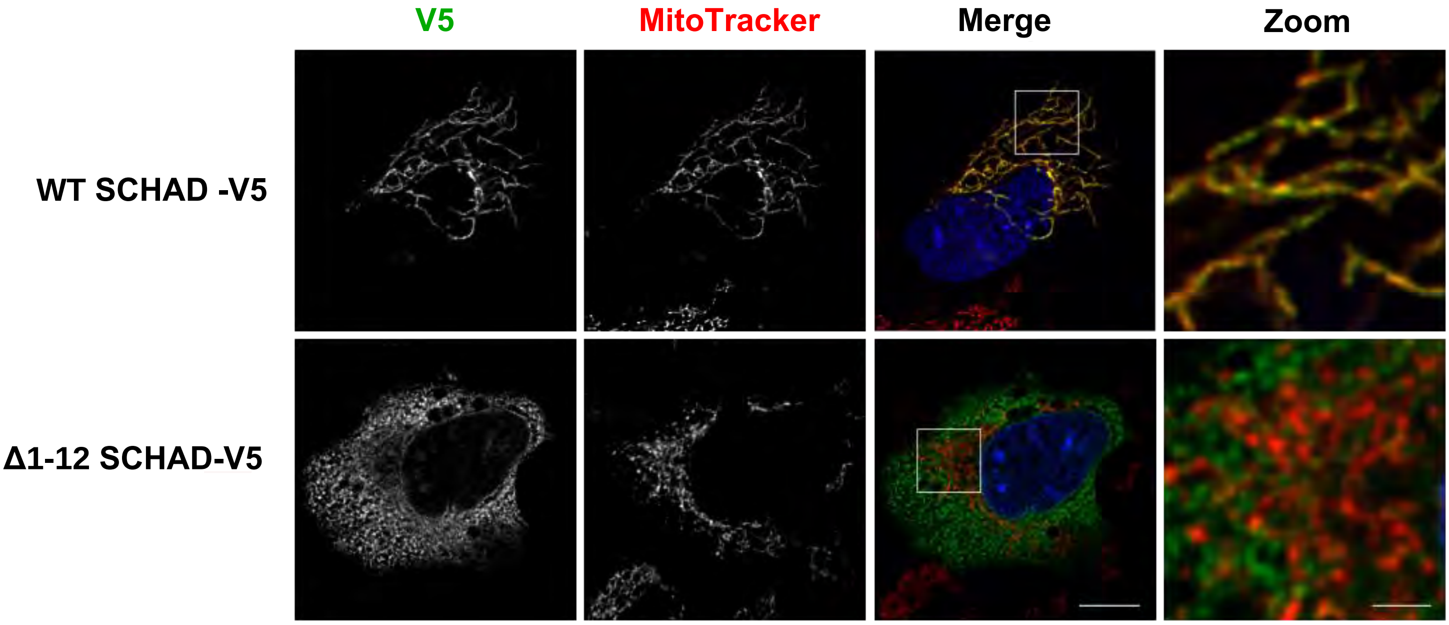

Supplementary Figure S3. Representative confocal image showing the effect of deleting the mitochondrial import signal in the SCHAD protein. HEK293 SCHADKO cells were transfected with V5-tagged SCHAD, either full length (WT) or lacking the import signal (Δ1-12). Cells were incubated with MitoTracker (red in the merged image), stained for SCHAD using an anti-V5 antibody (green), and nuclei counterstained with DAPI (blue). Scale bars = 10 µm (merge) and 2 µm (zoom). Three independent experiments were done with identical results.

Supplementary Figure S4

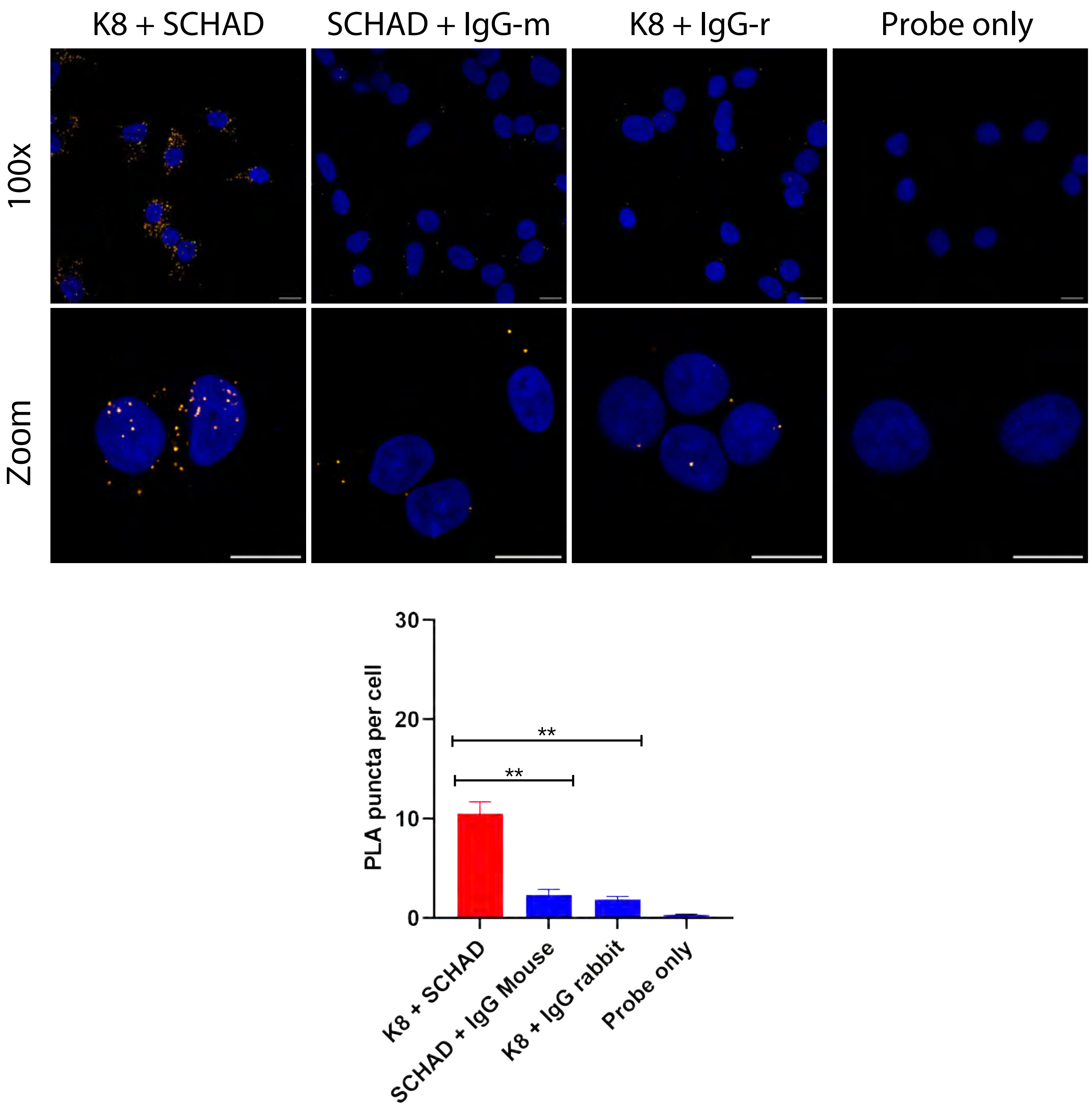

Supplementary Figure S4. Detection in EndoC-βH1 cells of interaction between the SCHAD and K8 proteins. EndoC-βH1cells grown in OPTIβ complete medium were investigated by a proximity ligation assay (PLA) using primary antibodies directed against SCHAD and K8. Negative controls were either a combination of anti-SCHAD and mouse IgG (IgG-m), or of anti-K8 and rabbit IgG (IgG-r). The probe-only control was completely negative. Scale bars = 10 µm. The graph shows quantification of the PLA puncta per cell. Data are presented as mean ± SEM. ** indicates $p<0.01$. 137 cells were quantified in total.

Uncropped images of all western blots

Figure 2B

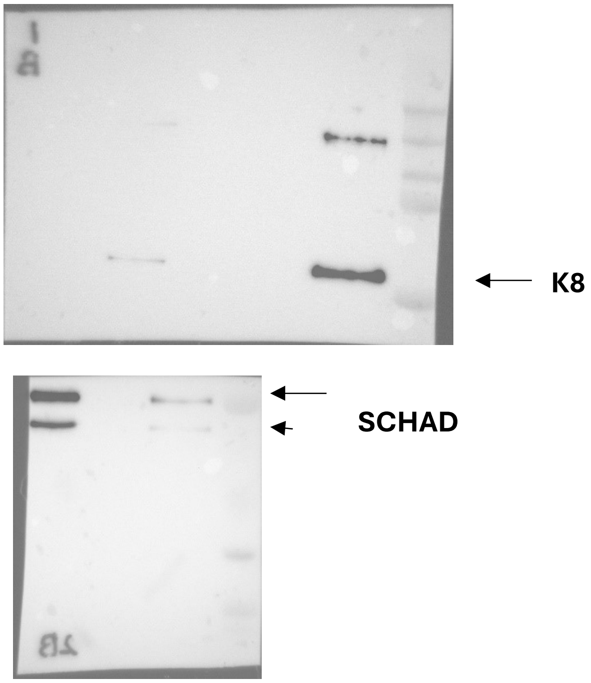

Figure 4A

Total pancreas

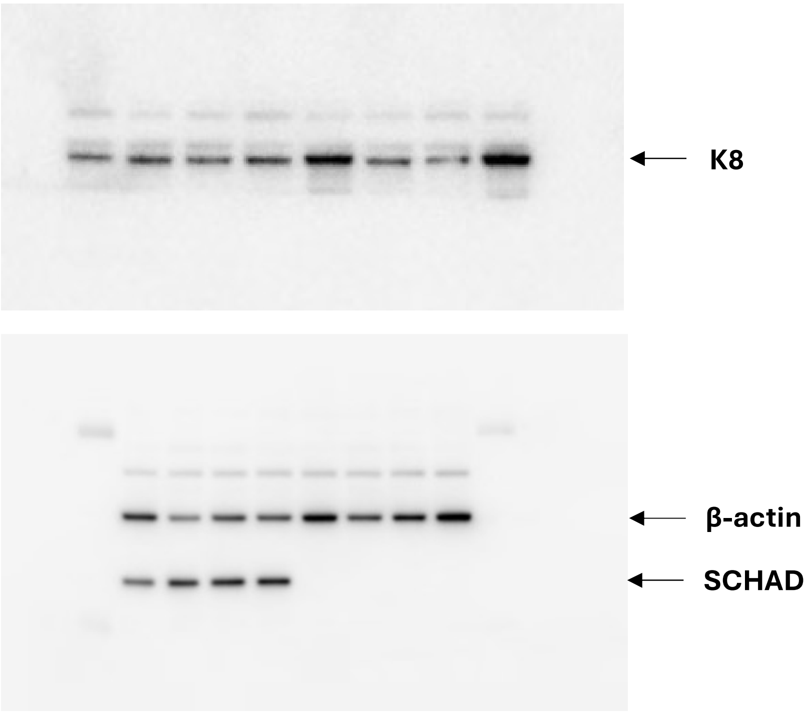

Figure 4A

Islets

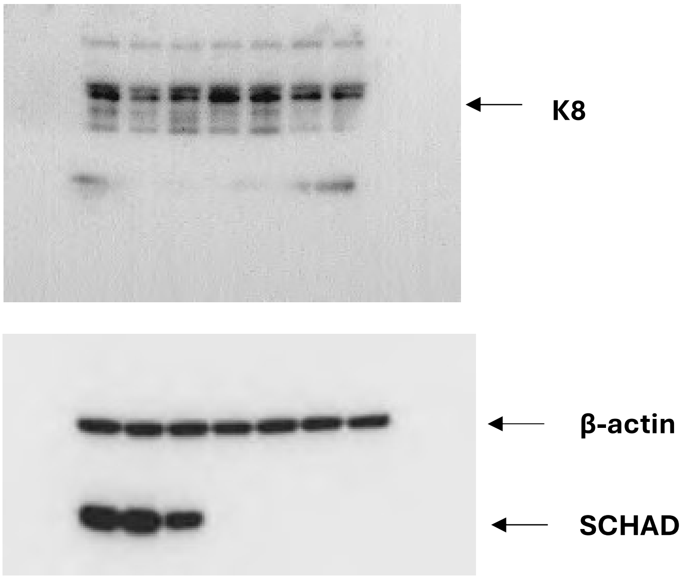

Figure 4B

Total pancreas

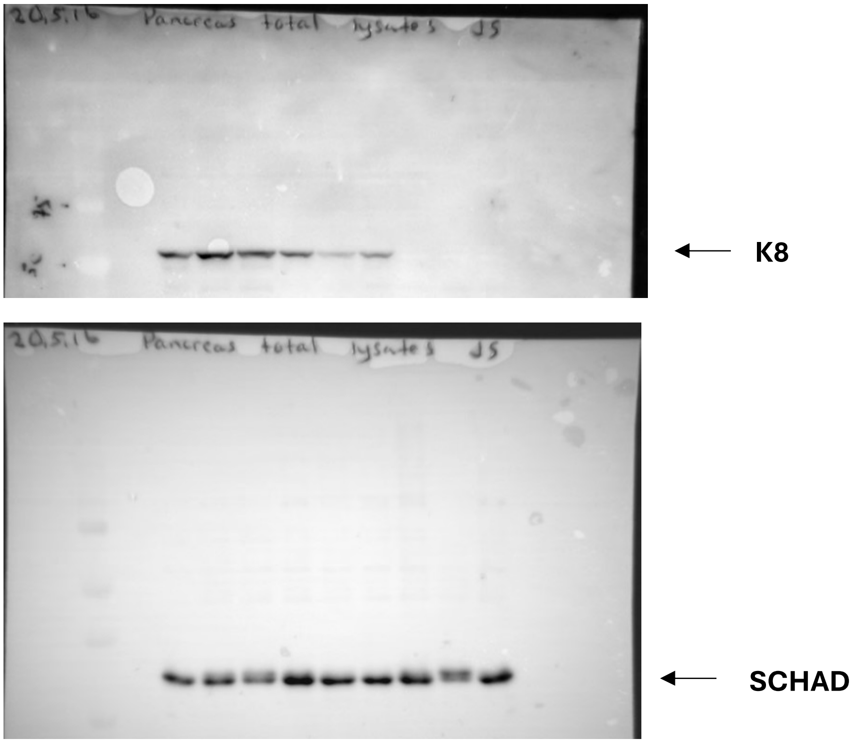

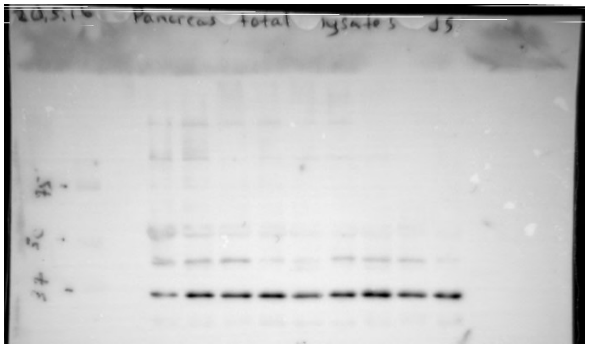

GAPDH

Figure 4B

Islets

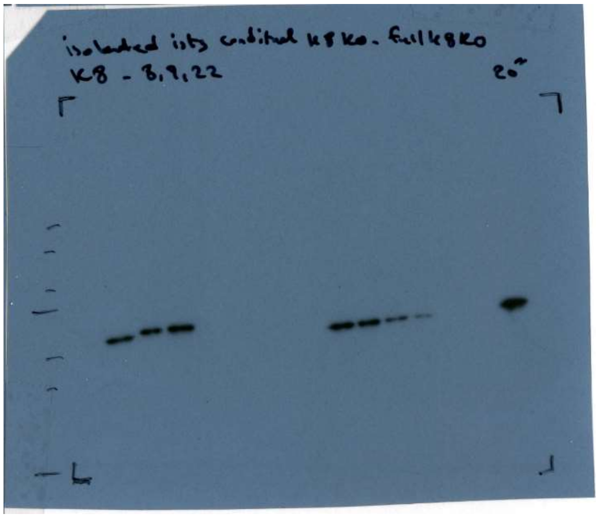

K8

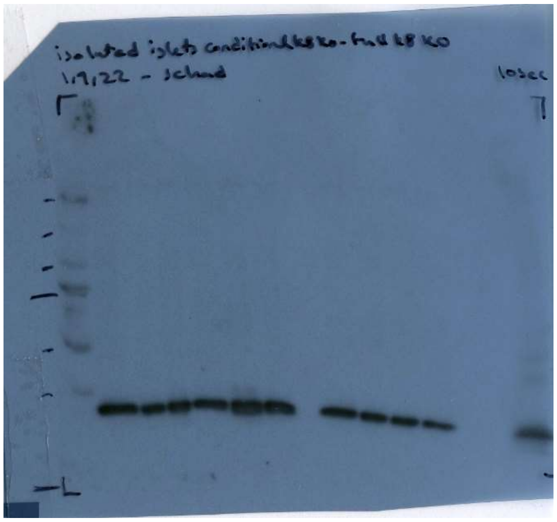

SCHAD

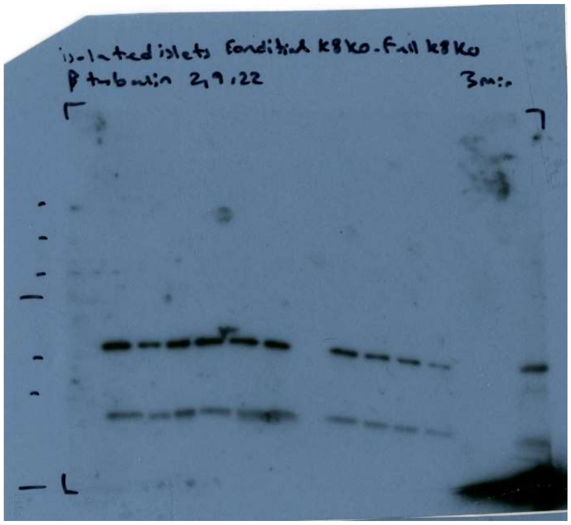

β-tubulin

Figure 5A

Males

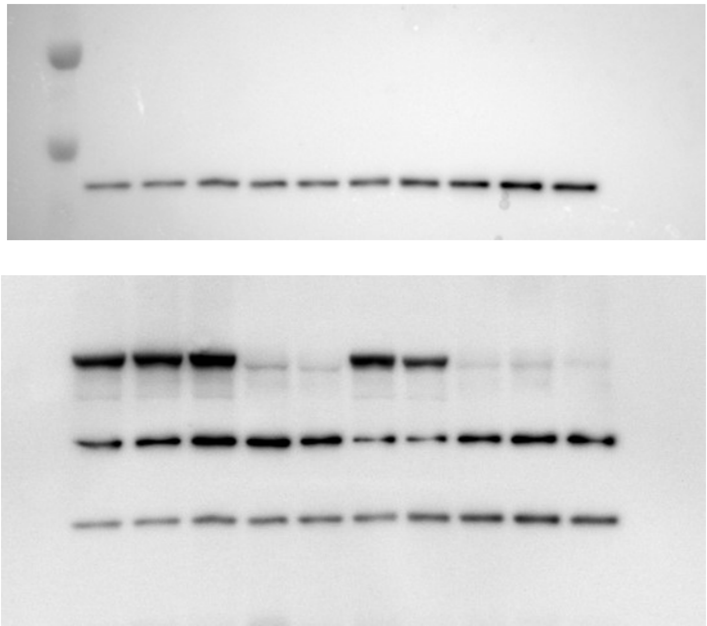

SCHAD

K8

β-actin

(SCHAD)

Figure 5B

Females

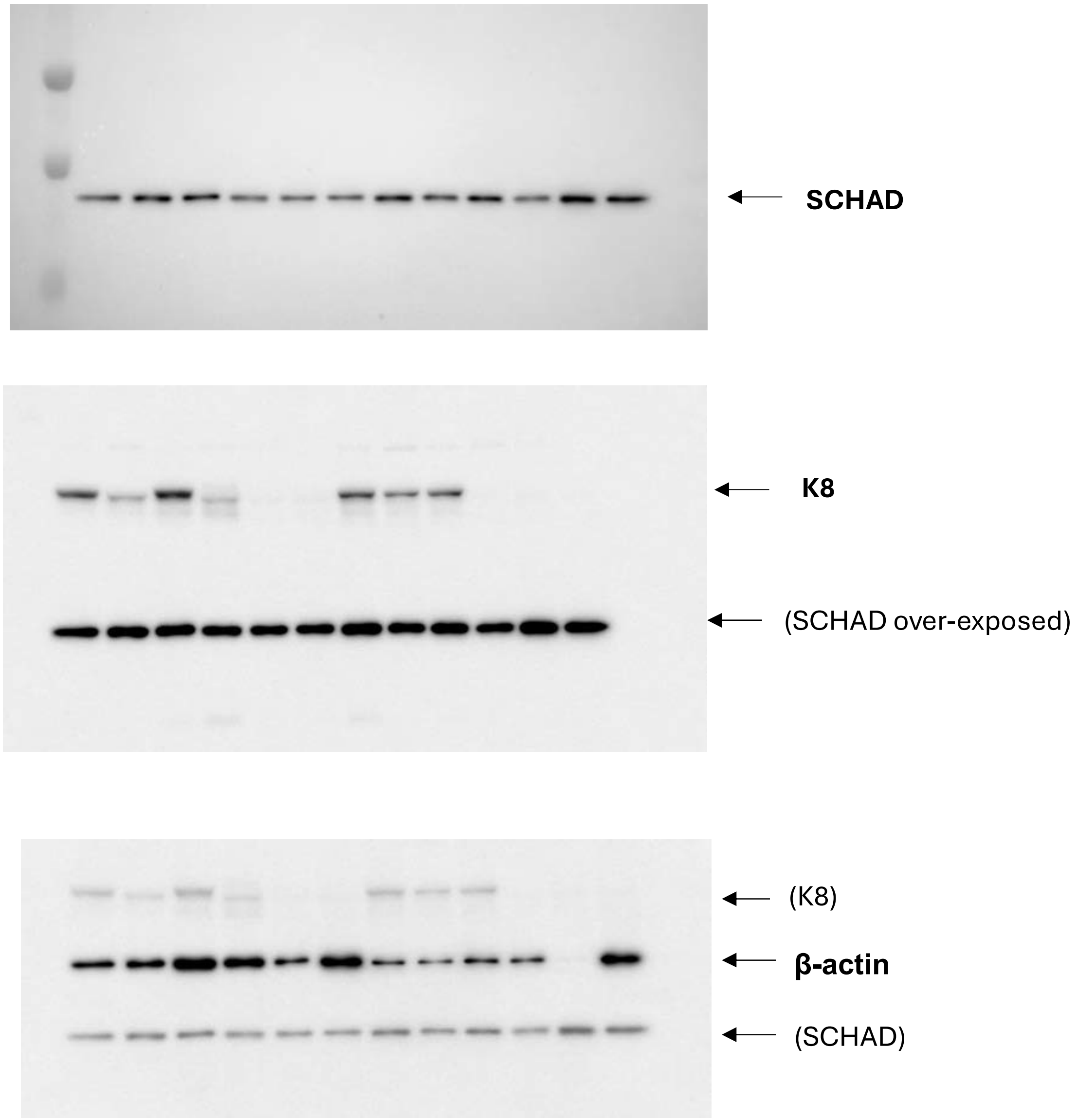

Supplement: Supplementary file 1 — Supplementary Material 1 [file 12860_2025_544_MOESM1_ESM.pdf]
